# *De Novo* Fatty Acid Synthesis During Mycobacterial Infection Is a Prerequisite for the Function of Highly Proliferative T Cells, But Not for Dendritic Cells or Macrophages

**DOI:** 10.3389/fimmu.2018.00495

**Published:** 2018-04-05

**Authors:** Philipp Stüve, Lucía Minarrieta, Hanna Erdmann, Catharina Arnold-Schrauf, Maxine Swallow, Melanie Guderian, Freyja Krull, Alexandra Hölscher, Peyman Ghorbani, Jochen Behrends, Wolf-Rainer Abraham, Christoph Hölscher, Tim D. Sparwasser, Luciana Berod

**Affiliations:** ^1^Institute of Infection Immunology, TWINCORE, Centre for Experimental and Clinical Infection Research, A Joint Venture Between the Medical School Hannover (MHH) and the Helmholtz Centre for Infection Research (HZI), Hannover, Germany; ^2^Infection Immunology, Research Center Borstel, Borstel, Germany; ^3^Core Facility Fluorescence Cytometry, Research Center Borstel, Borstel, Germany; ^4^Department of Chemical Microbiology, Helmholtz Centre for Infection Research, Braunschweig, Germany

**Keywords:** dendritic cells, macrophages, acetyl-CoA carboxylase 1, acetyl-CoA carboxylase 2, *Mycobacterium tuberculosis*, *Mycobacterium bovis* BCG, fatty acid synthesis, fatty acid oxidation

## Abstract

*Mycobacterium tuberculosis* (*Mtb*), the causative agent of human tuberculosis, is able to efficiently manipulate the host immune system establishing chronic infection, yet the underlying mechanisms of immune evasion are not fully understood. Evidence suggests that this pathogen interferes with host cell lipid metabolism to ensure its persistence. Fatty acid metabolism is regulated by acetyl-CoA carboxylase (ACC) 1 and 2; both isoforms catalyze the conversion of acetyl-CoA into malonyl-CoA, but have distinct roles. ACC1 is located in the cytosol, where it regulates *de novo* fatty acid synthesis (FAS), while ACC2 is associated with the outer mitochondrial membrane, regulating fatty acid oxidation (FAO). In macrophages, mycobacteria induce metabolic changes that lead to the cytosolic accumulation of lipids. This reprogramming impairs macrophage activation and contributes to chronic infection. In dendritic cells (DCs), FAS has been suggested to underlie optimal cytokine production and antigen presentation, but little is known about the metabolic changes occurring in DCs upon mycobacterial infection and how they affect the outcome of the immune response. We therefore determined the role of fatty acid metabolism in myeloid cells and T cells during *Mycobacterium bovis* BCG or *Mtb* infection, using novel genetic mouse models that allow cell-specific deletion of ACC1 and ACC2 in DCs, macrophages, or T cells. Our results demonstrate that *de novo* FAS is induced in DCs and macrophages upon *M. bovis* BCG infection. However, ACC1 expression in DCs and macrophages is not required to control mycobacteria. Similarly, absence of ACC2 did not influence the ability of DCs and macrophages to cope with infection. Furthermore, deletion of ACC1 in DCs or macrophages had no effect on systemic pro-inflammatory cytokine production or T cell priming, suggesting that FAS is dispensable for an intact innate response against mycobacteria. In contrast, mice with a deletion of ACC1 specifically in T cells fail to generate efficient T helper 1 responses and succumb early to *Mtb* infection. In summary, our results reveal ACC1-dependent FAS as a crucial mechanism in T cells, but not DCs or macrophages, to fight against mycobacterial infection.

## Introduction

*Mycobacterium tuberculosis* (*Mtb*), the causative agent of tuberculosis (Tb), remains a major health problem worldwide, a situation that becomes aggravated by increasing cases of multidrug-resistant strains. One of the main obstacles for the eradication of Tb is the enormous reservoir of chronically infected patients, estimated as up to two billion people. Of them, 5–10% will develop active disease. Consequently, a better understanding of the basic mechanisms employed by the pathogen to persist within the host is of major importance to design therapeutic strategies aiming at completely eliminating the bacteria. *Mtb* is usually transmitted *via* aerosol droplets. Once in the lungs, mycobacteria are recognized and phagocytosed by alveolar macrophages (AMs) and patrolling dendritic cells (DCs). AMs serve as a niche for initial bacterial replication, until these cells die by apoptosis or necrosis and mycobacteria spread to the extracellular space where they can be detected by other mononuclear cells. This initiates an inflammatory response that leads to the formation of the granuloma and containment of bacterial growth. Macrophages exert a pivotal role in this process through different microbicidal mechanisms (1), including nutrient restriction, the production of reactive oxygen and nitrogen species (ROS; RNS), and the induction of autophagy (1–3). Despite this, *Mtb* has acquired the capacity to persist in macrophages for long periods of time, exploiting the host cell machinery for its own purposes.

Emerging evidence suggests that *Mtb* pathogenicity is related to the manipulation of core metabolic pathways in the host cell. Under normal physiological conditions, immune cells are relatively quiescent and rely on the process of oxidative phosphorylation (OXPHOS) in the mitochondria to obtain energy for their housekeeping functions. Infection with *Mtb* leads to an induction in aerobic glycolysis as evidenced by high lactate levels and increased expression of glycolytic enzymes in the lungs of infected mice (4). Additionally, genome-wide transcriptional profiling of lung granulomas from patients with active Tb revealed increased activity of the glycolytic pathway (5). Aerobic glycolysis was first described in the 1920s by the German Nobel laureate Otto Warburg for tumor cells and refers to the conversion of glucose to lactate in the presence of oxygen. Although this process has long been attributed to highly proliferative cells, it has recently become evident that macrophages also make use of this metabolic pathway to sustain specific functions. For example, augmented glycolytic flux is a signature of classically activated “M1” macrophages (6, 7) and has also been observed in bone marrow-derived macrophages (BMDMs) and AMs upon infection with different *Mtb* strains (8–10). Engagement of the glycolytic pathway by *Mtb* results in increased lipid metabolism, thus promoting lipid body (LB) formation and differentiation into “foamy” macrophages, a hallmark of granulomas in patients with Tb (11, 12). LBs, consisting of triacylglycerols and sterol esters, may serve as a source of nutrients and building blocks for *Mtb*, as suggested by the finding that *Mtb* resides closely associated to LBs within macrophages (12). Strikingly, *Mtb* survival depends on these host lipids. Lipid accumulation in macrophages diminishes their mycobacterial killing capacity through inhibition of autophagy and lysosome acidification (13, 14). However, how lipid metabolism affects other macrophage functions remains unknown. Furthermore, the mechanisms by which *Mtb* induces LB formation and foam cell differentiation are not fully understood. Recent studies suggested that accumulation of LBs relies on the induction of *de novo* cholesterol and fatty acid synthesis (FAS) and the generation of the ketone body d-3-hydroxybutyrate by the host cell (9, 14). Moreover, while early-secreted antigenic target (ESAT-6), the main virulence factor of *Mtb*, has been identified as a main factor contributing to LB formation (9, 15), LBs can also be found in macrophages infected with avirulent *Mycobacterium bovis* BCG, suggesting diverse mechanisms behind this phenomenon (16).

In contrast to macrophages, DCs are not specialized in the killing of mycobacteria (17, 18), but instead are essential for the induction of adaptive immunity by transporting antigens to the lung draining lymph nodes, secreting inflammatory IL-12, and subsequently priming naïve T cells to become T helper 1 (Th1) cells (19, 20). The control of mycobacterial infection largely depends on these Th1 cells that secrete IFN-γ, and thereby promote mycobacterial control by activating macrophages (21). In accordance, depletion of CD11c^+^ DCs results in diminished generation of antigen-specific Th1 cells and increased bacterial burden (22). DCs are categorized into different subpopulations according to their function and localization, playing specific roles during mycobacterial infection. The involvement of certain DC subsets depends on the route of infection. Following intravenous (i.v.) infection, mycobacteria will be mostly encountered by CD8^+^ DCs that are present in the spleen. These CD8^+^ DCs mediate protective immunity by secreting IL-12 to induce IFN-γ production by Th1 cells (23–25) and by cross-presenting antigen to CD8^+^ T cells. Conversely, upon aerosol infection, most bacteria reside in the lungs, which contain only CD103^+^ DCs and CD11b^+^ DCs, but no CD8^+^ DCs (26). *Mtb*-resistant mouse strains (C57BL/6 and BALB/c) display higher numbers of CD103^+^ DCs in the lungs than the susceptible strain DBA/2 (27), suggesting that this subpopulation might be involved in mediating protection. However, the exact role of CD103^+^ DCs during mycobacterial infection remains unclear.

We previously showed that activation of DCs *via* the toll-like receptor (TLR)/MyD88 pathway is crucial for mycobacterial protection (28). In granulocyte-macrophage colony-stimulating factor (GM-CSF)-derived DCs, TLR/MyD88 ligation leads to a rapid metabolic reprogramming from OXPHOS toward aerobic glycolysis (29, 30). This early increase in glycolytic flux was proposed to support *de novo* FAS for the expansion of membranes in the ER and Golgi apparatus (30). Pharmacological inhibition of *de novo* FAS impairs GM-CSF DC cytokine production and activation upon TLR stimulation (30). Consequently, it has been suggested that engagement of this metabolic pathway might be relevant for full DC function. In contrast, in tumor-bearing mice and cancer patients, high lipid content in DCs led to functional impairment and uncontrolled tumor growth (31). However, the importance of this pathway *in vivo* has not yet been investigated and no studies on the role of FAS for the function of DCs during mycobacterial infection have been conducted.

Acetyl-CoA carboxylase (ACC) 1 and 2 (ACC1 and ACC2) are the rate-limiting enzymes for FAS and fatty acid oxidation (FAO). Both enzymes catalyze the same biochemical reaction, the ATP-dependent carboxylation of acetyl-CoA to malonyl-CoA. While ACC1 is located in the cytoplasm, ACC2 is associated with the outer mitochondrial membrane, where it controls FAO by regulating import of long chain fatty acids into the mitochondria *via* the carnitine palmitoyltransferase 1 (CPT1) (32). We have previously shown that during autoimmunity, ACC1 expression and *de novo* FAS are essential for the differentiation of naïve T cells toward inflammatory T effector lineages (33–35). In this study, we investigated the importance of ACC1 and ACC2 for DC, macrophage and T cell activation and their capacity to induce protective immunity against mycobacterial infection. Our results suggest that while ACC1 and ACC2 expression in DCs and macrophages is dispensable for mycobacterial control, T cells greatly depend on ACC1 and *de novo* FAS to cope with infection.

## Materials and Methods

### Mice

All mice were bred and kept under specific pathogen-free conditions at the animal facility of the Helmholtz Center for Infection Research (HZI, Braunschweig, Germany) or at TWINCORE (Hannover, Germany). C57BL/6 mice were purchased from Jackson Laboratories or bred in house. ACC1^*flox/flox*^ mice (36), Acaca*^flox/flox^* (37), or ACC2^*flox/flox*^ mice (38) were crossed to the following *cre*-expressing lines: CD4-*cre* mice (39) (TACC1), LysM-*cre* (40) (MΦ_ACC1 and MΦ_Acaca), or Itgax-*cre* (41) (DC_ACC1 and DC_Acaca) and maintained on a C57BL/6 genetic background. ACC2 knockout mice (42) were backcrossed to C57BL/6 background. Furthermore, P25ktk transgenic mice were used and bred in the same institutions (43). Sex- and age-matched littermates between 8 and 16 weeks of age were used for all experiments.

### Mycobacterial Infections

*Mycobacterium bovis* BCG overexpressing Ag85B (*M. bovis* BCG Ag85B) was kindly provided by Dr. Joel Ernst (NYU School of Medicine, USA), *M. bovis* BCG GFP by Dr. Camille Locht (University of Lille), and *M. bovis* BCG RFP by Dr. Nathalie Winter (French National Institute for Agricultural Research). All strains were grown at 37°C in Middlebrook 7H9 broth (BD Biosciences) supplemented with 10% Middlebrook OADC enrichment medium (BD Biosciences), 0.002% glycerol (Roth), and 0.05% Tween 80 (Roth). Midlog phase cultures were harvested, aliquoted, and frozen at −80°C. For *in vitro* infections BCG strains were prepared as previously described (44) and cells infected with different multiplicities of infection (MOI). Bacteria for *in vivo* infections were prepared from frozen stocks by thawing at 37°C, washing with PBS 0.025% Tween 80 (PBS-T), and passaging through a 27 gauge needle. Mice were infected intravenously (i.v.) with 2 × 10^6^ colony forming units (CFU). For *Mtb* infections, the *Mtb* strain H37Rv was grown and prepared as described previously (45). Mice were infected with a low (100 CFU) or a high dose (1,000 CFU) by aerosol exposure. *Mtb*-infected mice were scored according to Morton and Griffiths (46). According to the local animal welfare guidelines, mice that reached a score of >3.0 had to be euthanized.

### Colony Enumeration Assay

To determine CFU, mice were sacrificed 21 days p.i. (for *M. bovis* BCG) or at different time points p.i. (for *Mtb*). Liver, spleen, and lungs were removed and organs were plated in serial dilutions as described previously (28, 45). CFU were enumerated after incubation at 37°C for 3 weeks. Data are presented as log_10_ CFU per organ.

### Generation of iCD103 DCs, GM-CSF DCs, and BMDMs

Dendritic cell and macrophage cultures were started from BM cells, which were isolated from murine femurs and tibiae. iCD103 DCs were generated as described previously (47). In brief, BM cells were cultured in complete RPMI (cRPMI) 1640 GlutaMAX medium (Thermo Fisher Scientific), supplemented with 10% heat-inactivated FCS (Biochrom), 500 U penicillin-streptomycin (PAA laboratories), and 50 μM β-mercaptoethanol (Thermo Fisher Scientific) with a combination of GM-CSF and FLT3L (both self-made) for 9 days, followed by re-plating with both growth factors for additional 7 days at 37°C with 5% CO_2_. For generating GM-CSF DCs, BM cells were cultured in cRPMI supplemented with 5% culture supernatant of a GM-CSF-producing cell line (48). For generating BMDMs, BM cells were incubated in cRPMI supplemented with L929 cell conditioned medium (LCCM; self-made) as a source of murine M-CSF for 7 days. After 3 days, half of the medium was replenished by fresh medium containing LCCM. Flt3-L-producing CHO Flt3-L FLAG cells were generated by Dr. Nicos Nicola and kindly provided by Dr. Karen Murphy (WEHI, Melbourne, VIC, Australia). The LCCM producing L929 cell line was kindly provided by Dr. Roland Lang (Universitätsklinikum Erlangen, Erlangen, Germany).

### *In Vitro* Infection and Activation of iCD103 DCs and BMDMs

iCD103 DCs and BMDMs were harvested on day 16 or 7, respectively. A gradient with Optiprep (Progen Biotechnik) was performed to deplete the cell suspensions from dead cells. Cells were stimulated with CpG-B 1826 (1 μM; TIB MOLBIOL) or LPS (100 ng/mL, *E. coli* Serotype 055:B5; Sigma) or infected with the BCG strains mentioned above at different MOIs. Infection was monitored by evaluating the frequency of GFP^+^ or RFP^+^ cells by flow cytometry or confocal microscopy. As a positive control of complete blockade of ACC activity, cells were treated with SorA (1 μM; kindly provided by Dr. Rolf Müller, Helmholtz Institute for Pharmaceutical Research Saarland) or 5-(Tetradecyloxy)-2-furoic acid (TOFA) (20 μM; Enzo Life Sciences) during stimulation.

### Flow Cytometry

The following monoclonal antibodies specific to mouse antigens and labeled with the indicated fluorescent markers were purchased from eBioscience/Thermo Fisher Scientific: CD3e eFluor450 (17A2), CD19 eFluor450 (eBio1D3), CD4 eFluor450 (RM4-5), CD4 Alexa488, CD4 eFluor660, CD4 PE-Cy7 (all GK1.5), CD8a (Ly-2) FITC, CD8a (Ly-2) eFluor450 (both 53-6.7), CD45.1 APC (A20) FoxP3 eFluor450, FoxP3 PE (both FJK-16s), CD62L PE-Cy7, CD62L PE, CD62L APC-eFluor780 (all MEL-14), IL-17A APC, IL-17A PE-Cy7 (both eBio17B7), IFN-γ FITC, IFN-γ PE, IFN-γ PE-Cy7 (all XMG1.2), CD11c (Integrin alpha chain) eFluor660, CD11c PE, CD11c APC-eFluor780 (all N418), CD11b PE, CD11b FITC (both M1/70), NK1.1 eFluor450 (PK136), CD45R/B220 PE-Cy7, CD45R/B220 PE (both RA3-6B2), CD86 (I-A/I-E) APC, CD86 (I-A/I-E) FITC (both GL1), MHC-II FITC, MHC-II eFluor450 (both M5/114.15.2), F4/80 eFluor450, F4/80 eFluor660 (both BM8), TCRbeta APC-eFluor780 (H57-597), CD90.2 APC-e780 (53-2.1), rat IgG1, κ isotype control FITC, rat IgG2a, κ isotype control FITC, rat IgG2b, and κ isotype control eFluor450. IL-10 PE (JES5-16E3), CD103 PB (2E7), CD44 FITC (IM7), and IFN-γ (XMG1.2) were purchased from Biolegend and CD4 V450 (RM4-5), CD25 APC (PC61), and CD62L PE (MEL-14) were purchased from BD. The I-A^b^ ESAT-6 (4-17) APC tetramer was provided by the NIH Tetramer Core Facility, Emory University Vaccine Center, Atlanta, GA, USA. To analyze intracellular cytokine production by T cells *ex vivo*, cells were stimulated with phorbol 12-myristate 13-acetate (PMA, 100 ng/mL; Sigma-Aldrich) and ionomycin (1 μg/mL; Sigma-Aldrich) for 2 h followed by additional 2 h with Brefeldin A (5 μg/mL; eBioscience/Thermo Fisher Scientific). Where indicated, cells were alternatively stimulated with 5 μg/mL plate-bound anti-CD3/CD28 (clones 145-2C11 and 37.5, respectively; BD). To analyze intracellular cytokine production by iCD103 DCs and BMDMs, cells were stimulated or infected for a total of 6 h and incubated with Brefeldin A (5 μg/mL; eBioscience/Thermo Fisher Scientific) for the last 4 h. Intracellular staining was performed after fixation with paraformaldehyde (2%; Roth) and permeabilization with PBA-S buffer (0.5% Saponin and 0.25% BSA in PBS; both Roth). For FoxP3 staining, the FoxP3/Transcription Factor Fixation/Permeabilization Kit (eBioscience/Thermo Fisher Scientific) was used according to the manufacturer’s instruction. For assessing DC activation *in vivo*, spleens were digested using Collagenase D (500 μg/mL; Roche) and DNase I (50 μg/mL; Roche) for 30 min at 37°C. The uptake of palmitate was determined by incubating cells in 100 μL PBS with 1 μg/mL Bodipy FL C_16_ (Thermo Fisher Scientific) at 37°C for 30 min.

The accumulation of lipids was evaluated using HCS LipidTOX™ Phospholipidosis and Steatosis Detection Kit (catalog number H34158; Thermo Fisher Scientific), which contains LipidTOX™ Red phospholipid stain and LipidTOX™ Green neutral lipid stain. For the detection of phospholipids, LipidTOX™ Red was added to the cells together with the stimuli or bacteria. For assessing the accumulation of neutral lipids, cells were stained with LipidTOX™ Green neutral lipid stain after treatment and PFA fixation according to the manufacturer’s instructions. Data acquisition was conducted on a CyAn ADP (Beckman Coulter) or a LSR II (Becton Dickinson), and data were analyzed with FlowJo software (Tree Star).

### *In Vitro* T Cell Proliferation Assay

iCD103 DCs or BMDMs were infected with different MOIs of *M. bovis* BCG Ag85B for 24 h in the presence or absence of SorA, washed four times and incubated at a 1:6 ratio (25,000 DCs/BMDMs:150,000 T cells) with naïve (CD4^+^CD25^−^) T cells obtained from spleen and lymph nodes of P25ktk mice. Naïve cells were enriched by negative magnetic cell sorting using the Dynal Mouse CD4 negative T cell isolation kit following the manufacturer’s protocol (Thermo Fisher Scientific). CD25^+^ cells were depleted by including an anti-CD25 functional grade antibody (clone PC61.5; eBioscience/Thermo Fisher Scientific) to the antibody cocktail for negative selection. After enrichment, cells were labeled with CellTrace Violet Cell Proliferation Kit (Thermo Fisher Scientific), as per manufacturer’s instructions. For Th17 cell induction, cells were cultured in complete IMDM GlutaMAX medium (Thermo Fisher Scientific) containing rhTGF-β1 (2 ng/mL; Peprotech), anti-IFN-γ (5 μg/mL, clone XMG1.2; Bio X Cell), anti-IL-4 (5 μg/mL, clone 11B11; Bio X Cell), and anti-IL-12 (5 μg/mL, clone 17.8; Bio X Cell). For Th0 conditions, cells were cultured in plain cRPMI. Soluble CD3 (1 μg/mL, clone 145-2C11; Bio X Cell) or P25 peptide (10 μg/mL; Department of Chemical Biology, HZI Braunschweig) were used as positive controls. Co-cultures were performed for 4 days in 96-well round bottom (Th0) or flat bottom plates (Th17). Proliferation and cytokine production were determined by intracellular flow cytometry staining.

### *In Vivo* T Cell Priming

WT or DC_ACC1 mice were infected with *M. bovis* BCG as described above. On day 9 p.i., 2–3 × 10^6^ CellTrace Violet labeled CD4^+^ T cells from P25ktk x CD45.1 mice were adoptively transferred i.v. After 5 days, mice were sacrificed and proliferation as well as cytokine production was determined upon PMA/ionomycin re-stimulation by intracellular flow cytometry staining.

### ESAT6_1–20_-Specific ELISPOT Assay

For measuring the frequency of antigen-specific CD4^+^ T cells after *Mtb* infection, single cell suspensions from lungs were prepared and collected in complete IMDM. Lung cells were stimulated for 20 h with mitomycin-D (Sigma-Aldrich)-inactivated spleen cells from uninfected mice that had been pulsed with the MHC class II peptide ESAT-6_1–20_ (Research Center Borstel). Detection of antigen-specific IFN-γ-producing CD4^+^ T cells was conducted using ELISPOT assay kits as described by the manufacturer (BD Bioscience and R&D Systems, respectively). Spots were automatically enumerated using an ELISPOT reader (ELISPOT 04 XL; AID) and the frequency of cytokine-producing cells was determined.

### Incorporation Assays

For ^13^C incorporation analysis, [U-^13^C_6_] glucose (1 mM; Cambridge Isotope Laboratories) or [U-^13^C_16_] palmitate (1 μM; Cambridge Isotope Laboratories) was added at the onset of the *in vitro* infection experiments. To determine the incorporation of glucose- or palmitate-derived carbon into cellular fatty acids, cells were saponified [MeOH:NaOH (15%) 1:1, 1 h, 100°C], derivatized [MeOH:HCl 10:2, 10 min, 80°C] and then prepared for analysis on a gas chromatography-combustion-isotope ratio mass spectrometer (GC/C/IRMS) as described earlier (49). GC/C/IRMS measurements were performed in triplicate on a Finnigan MAT 253 isotope ratio mass spectrometer coupled with a Trace GC Ultra (Thermo Fisher Scientific) chromatograph *via* a combustion interface. The fatty acid methyl esters were separated with an Optima five column (5% phenyl, 95% dimethylpolysiloxane, 50 m, 0.32 mm inner diameter, and 0.25 μm film thickness). The oven program was 100°C for 2 min, increased to 290°C at 4°C min^−1^, followed by an isothermal period of 8 min. The separated compounds were combusted on line in an oxidation oven. ^13^C/^12^C isotope ratios for the free fatty acids were calculated as described (49) and are presented as δ^13^C in the figures.

### Confocal Microscopy

iCD103 DCs and BMDMs were infected with *M. bovis* BCG GFP to check the infection rate. After 24 h, Hoechst 33342 was used to stain nuclei and cells were loaded in VI 0.5 μ-slides (Ibidi). Confocal microscopic images were taken on an Olympus FV1000 system using a 60× oil objective. All images were equally adjusted using Fiji software (NIH).

### ELISA

Supernatants from iCD103 DCs and BMDM infected with different MOIs of *M. bovis* BCG were taken 4 or 24 h p.i. Serum samples from infected mice were collected by heart puncture on the day of analysis. The concentration of IL-12/23p40, IL-6, TNF-α, IL-10, IL-1β, and IFN-γ was determined by ELISA according to the manufacturer’s instructions (Duo Set; R&D).

### Nitrite Assay

Nitrite levels were determined in culture supernatants from iCD103 DCs and BMDMs after 24 h of stimulation as an indicator of nitric oxide (NO) production using the Griess reagent system (Promega), as per manufacturer’s instructions.

### Cell Sorting

Mice were euthanized by CO_2_ inhalation. For alveolar macrophage isolation, mice were perfused with 5 mL PBS. Lung lobes were separated from the trachea, chopped, and incubated in digestion media, containing RPMI 1640 GlutaMAX medium (Thermo Fisher Scientific) supplemented with 5% FCS (Biochrom), containing 2.2 mg/mL collagenase D (Sigma-Aldrich) and 0.055 mg/mL DNase I (Roche) for 30 min at 37°C. For splenic DCs and macrophages, spleens were minced and incubated in digestion media for 30 min at 37°C. Digestion was stopped by addition of 10 mM ethylenediaminetetraacetic acid (EDTA), and the cell suspensions were passed through a 70 μm cell strainer. A gradient with Optiprep (Progen Biotechnik) was performed to deplete the cell suspensions of dead cells and erythrocytes. For isolation of peritoneal macrophages, 5 mL of PBS with 2% FCS (Biochrom) and 2 mM EDTA were injected into the peritoneal cavity and recovered. This process was repeated twice to obtain a final volume of 10 mL. For sorting of T cells, spleen and lymph nodes were isolated and homogenized. CD4^+^ T cells were sorted after enrichment using the Dynal Mouse CD4 negative T cell isolation kit following the manufacturer’s protocol (Thermo Fisher Scientific).

Cell suspensions were stained with the antibodies described above and the sorting strategy can be found in Figure S4 in Supplementary Material. For sorting myeloid cells from spleen and peritoneum, a lineage cocktail containing anti-CD3e, anti-CD19, and anti-NK1.1 conjugated to eFluor450 (eBioscience) was used. Dead cells were excluded using DAPI.

### Targeting Efficiency

iCD103 DCs, GM-CSF DCs, and BMDMs were lysed in TRIzol reagent (Thermo Fisher Scientific) and RNA was isolated with Direct-zol RNA MiniPrep (Zymo Research). In order to assess the rate of ACC1 deletion *in vivo*, macrophages and DCs were sorted from MΦ_ACC1 or DC_ACC1 mice directly into RLT buffer (Qiagen). RNA was isolated with the RNeasy Micro Kit (Qiagen) following the manufacturer’s instructions. RNA quality and concentration was determined with a 2100 Bioanalyzer (Agilent Technologies). RNA from *in vitro*-cultured and *ex vivo*-isolated cells was retrotranscribed into cDNA using SuperScript III Reverse Transcriptase Kit (Thermo Fisher Scientific). Real-time PCR reactions were carried out in a StepOne Real-time PCR system (Thermo Fisher Scientific) using Fast SYBR Green Master Mix (Thermo Fisher Scientific). *Acc1* mRNA levels were normalized to the housekeeping gene *Actb*.

### Statistical Analysis

Data analyses were performed using GraphPad Prism Software version 6.0 (GraphPad Software) and statistics were calculated using Student’s *t*-test, one-way ANOVA with Dunnett’s correction or two-way ANOVA with Bonferroni correction. *P*-values were considered significant as follows: *P < 0.05 and **P < 0.01, ***P < 0.001, ****P < 0.0001.

## Results

### iCD103 DCs Display Low Infection Rates but Become Highly Activated in Presence of Mycobacteria

**Figure 1:**
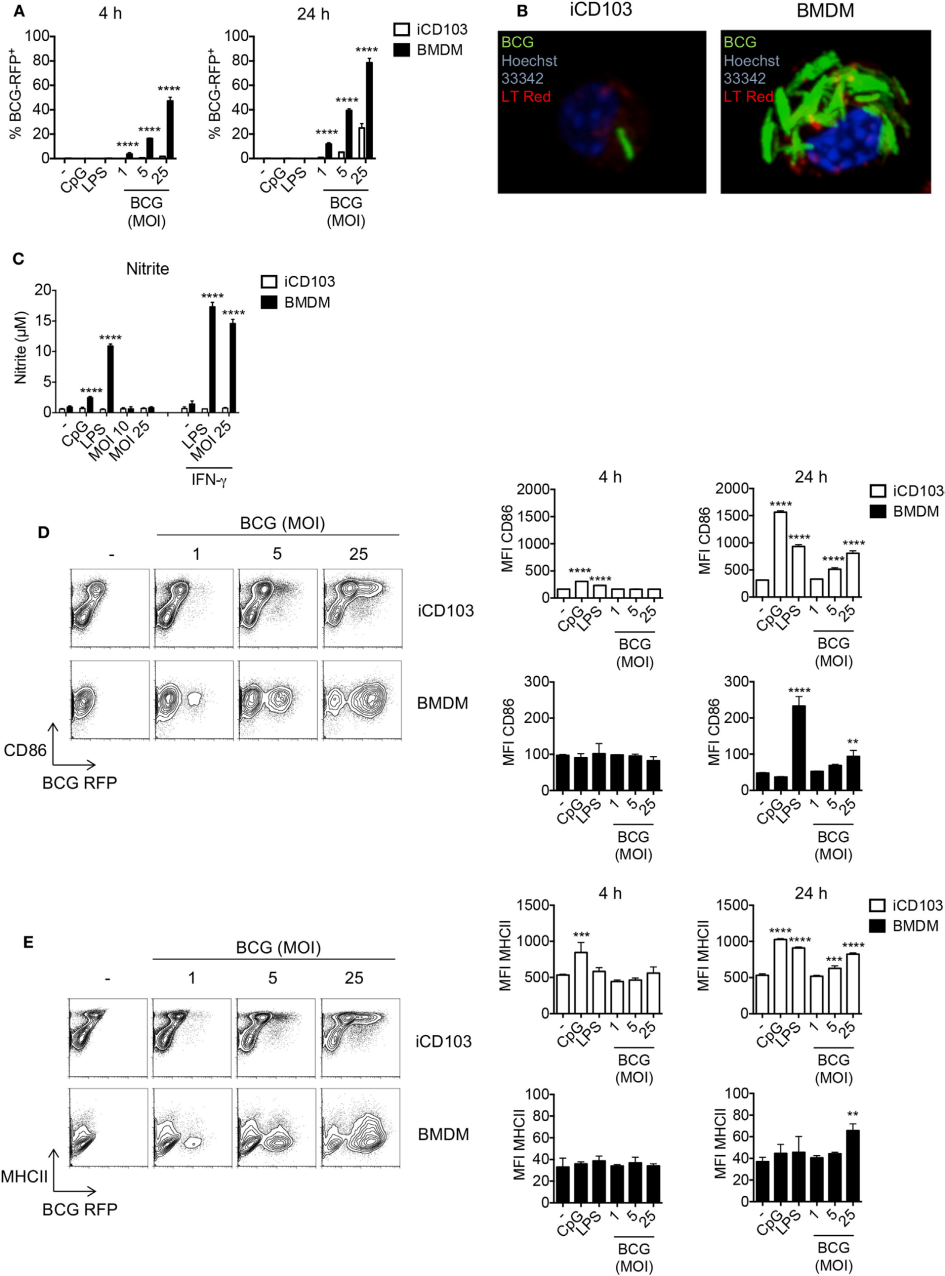
iCD103 dendritic cells (DCs) display low infection rates, but become highly activated in the presence of mycobacteria. iCD103 DCs and bone marrow-derived macrophages (BMDMs) were infected with different multiplicities of infection (MOIs) of *Mycobacterium bovis* BCG RFP or GFP and the infection rate, nitrite production, as well as activation status was analyzed after 4 and 24 h. LPS and CpG were used as stimulation controls. **(A)** Graphs show frequency of RFP^+^ iCD103 DCs and BMDMs among live cells. **(B)** Confocal microscopy images of iCD103 DCs and BMDM at 24 h p.i. with MOI 10 of *M. bovis* BCG GFP (green) and stained for nuclei (Hoechst 33342; blue) and phospholipids (LT Red; red). **(C)** Bar graphs show nitrite levels in culture supernatants after 24 h determined by Griess reagent system. IFN-γ served as a positive control. **(D)** Representative flow cytometry plots display CD86 expression and *M. bovis* BCG RFP in iCD103 DCs and BMDMs at 24 h p.i. (left panel). Bar graphs show the MFI of CD86 expression at 4 and 24 h p.i. (right panel). **(E)** Representative flow cytometry plots display MHCII expression and *M. bovis* BCG RFP in iCD103 DCs and BMDMs at 24 h p.i. (left panel). Bar graphs show the MFI of MHCII expression at 4 and 24 h p.i. (right panel). Results are representative of two [**(B)**, BMDM], **(C)**, three [**(B)**, iCD103 DC], five **(D,E)**, or six **(A)** experiments. Error bars represent SD of triplicates. *P < 0.05, **P < 0.01, ***P < 0.001, ****P < 0.0001, two-way ANOVA with Bonferroni correction **(A,C)**, or one-way ANOVA with Dunnett’s correction **(D,E)**.

Early studies investigating the role of DCs and macrophages during mycobacterial infection are mainly based on *in vitro*-generated macrophages and GM-CSF DCs derived from murine bone marrow (BM) (17). Yet, GM-CSF DCs do not resemble the DC populations present in the lung which mainly consist of CD11b^+^ and CD103^+^ DCs. Recently, pulmonary CD103^+^ DCs were shown to be important for the transport of mycobacteria to the lung-draining lymph nodes where they induce T cell responses (50). Thus, to explore the effect of mycobacteria on CD103^+^ DCs, we made use of a novel method to generate large numbers of CD103^+^ DCs (iCD103 DCs) *in vitro* functionally resembling lung CD103^+^ DCs (47). By means of this system, we first determined the rate of infection in iCD103 DCs by flow cytometry using RFP-expressing *M. bovis* BCG and compared it to BMDMs. Our results show that the percentage of RFP^+^ BMDMs increases rapidly after infection, with almost 50% of the cells being infected after 4 h (MOI 25). This rate further increased up to 80% after 24 h. In contrast, no RFP^+^ iCD103 DCs could be detected after 4 h of infection and only 30% of them were RFP^+^ 24 h post infection (p.i.) with the highest MOI tested (Figure 1A). Confocal analysis using GFP*-*expressing *M. bovis* BCG revealed that the majority of the bacteria were present within the cells, suggesting that BMDMs have a higher internalization capacity for mycobacteria than iCD103 DCs (Figure 1B). To rule out the contribution of microbicidal mechanisms to the low infection rates observed in iCD103 DCs, we measured the accumulation of nitrite (${\text{NO}}_{2}^{-}$) in the culture supernatants as an indicator of NO production (Figure 1C). As expected, iCD103 DCs were not able to produce ${\text{NO}}_{2}^{-}$ upon infection or TLR stimulation. In contrast, BMDMs produced high levels of ${\text{NO}}_{2}^{-}$ upon infection with *M. bovis* BCG, which were dependent on the presence of IFN-γ. Thus, we could confirm that the low frequency of infection observed for iCD103 DCs is not a result of increased killing capacity.

Exposure of DCs and macrophages to mycobacteria or their products triggers TLR-signaling, which subsequently leads to their activation, upregulation of costimulatory molecules, and production of inflammatory cytokines required for bacterial containment (51). Therefore, we next evaluated the activation status of iCD103 DCs and BMDMs upon *M. bovis* BCG infection or stimulation with the TLR ligands CpG (TLR9) or LPS (TLR4) by assessing the expression of CD86 and MHC class II (MHCII). In iCD103 DCs, LPS and CpG led to a minor upregulation of CD86 and MHCII already 4 h after stimulation and this expression strongly increased after 24 h. The effect of *M. bovis* BCG was less pronounced and only observed after 24 h with a MOI ≥ 5 (Figures 1D,E). In contrast, BMDMs displayed no changes at 4 h in all conditions tested and only increased CD86 expression 24 h after stimulation with LPS or MOI 25 of *M. bovis* BCG (Figure 1D). Along the same line, MHCII expression on BMDMs increased only slightly upon infection with a MOI 25 of *M. bovis* BCG (Figure 1E). As expected with regards to their pivotal role in presenting antigens to T cells, the expression of MHCII and CD86 was in general much higher in iCD103 DCs than in BMDMs, even when the cells were not stimulated.

### iCD103 DCs Produce High Levels of IL-12/23p40 and Have a Strong T Cell Priming Capacity Upon Mycobacterial Infection

**Figure 2:**
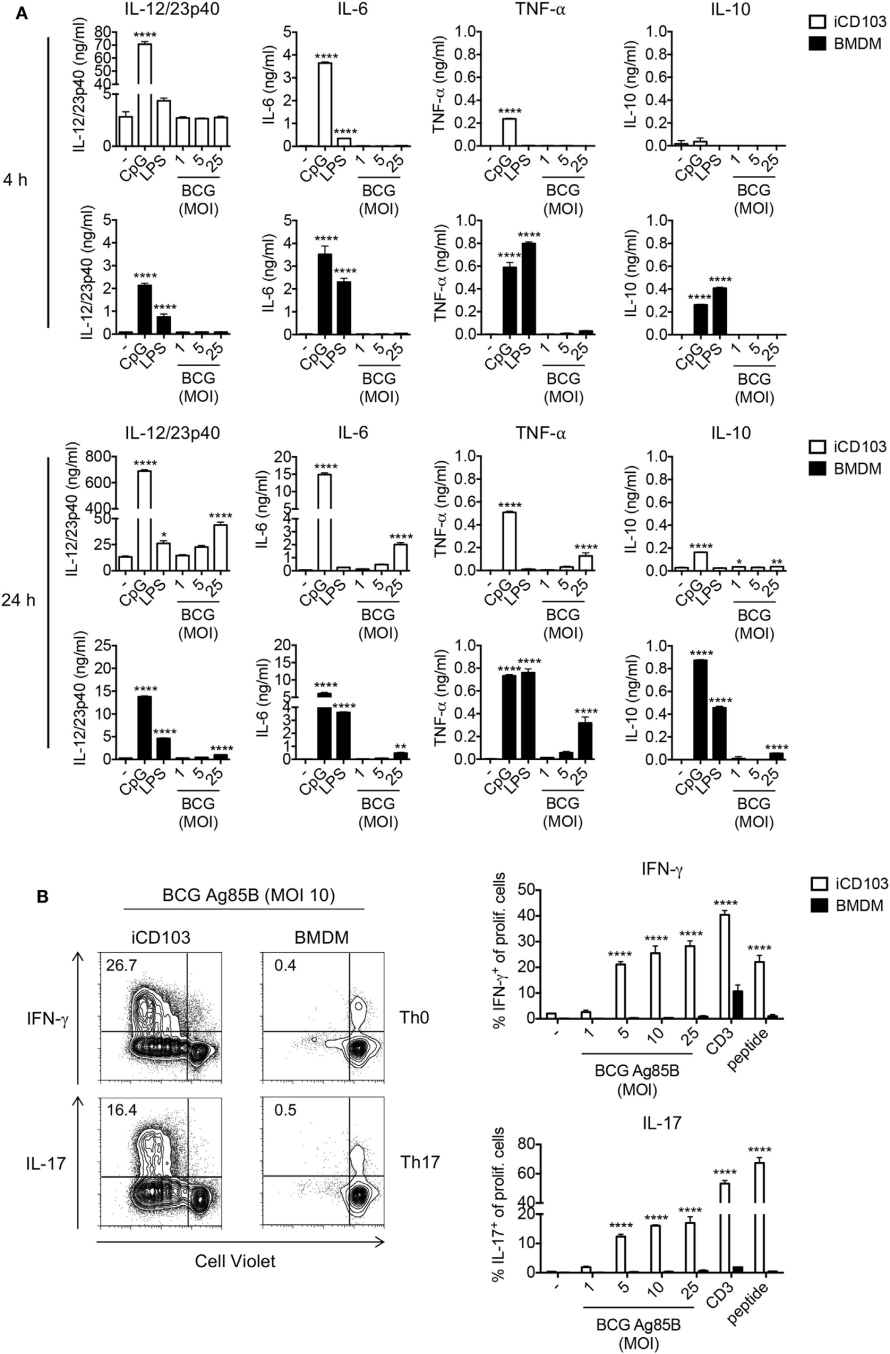
iCD103 dendritic cells (DCs) produce high levels of IL-12/23p40 and have a strong T cell priming capacity upon mycobacterial infection. **(A)** iCD103 DCs and bone marrow-derived macrophages (BMDMs) were infected with different multiplicities of infection (MOIs) of *Mycobacterium bovis* BCG RFP and levels of IL-12/23p40, IL-6, TNF-α, and IL-10 production were measured at 4 and 24 h p.i. by ELISA. LPS and CpG were used as stimulation controls. **(B)** iCD103 DCs or BMDMs were infected with *M. bovis* BCG overexpressing Ag85B and co-cultured with naïve CD4^+^CD25^−^ P25ktk T cells for 4 days under Th0 or Th17 polarizing conditions. Representative flow cytometry plots display proliferation and IFN-γ or IL-17 production of P25ktk cells upon infection of iCD103 DCs or BMDMs with MOI 10 of *M. bovis* BCG Ag85B (left panel). Bar graphs display the frequencies of IFN-γ^+^ or IL-17^+^ cells among total live CD4^+^ T cells upon re-stimulation with PMA/ionomycin (right panel). Results are shown as a representative of two individual experiments **(A,B)**. Error bars represent SD of triplicates. *P < 0.05, **P < 0.01, ***P < 0.001, ****P < 0.0001, one-way ANOVA with Dunnett’s correction **(A)**, or two-way ANOVA with Bonferroni correction **(B)**.

We next determined the cytokine profile of iCD103 DCs and BMDMs infected with *M. bovis* BCG by ELISA (Figure 2A). Upon TLR stimulation, both iCD103 DCs and BMDMs produced IL-12/23p40, IL-6, and TNF-α already 4 h after stimulation, whereas induction of cytokines by *M. bovis* BCG did not occur until 24 h p.i. (Figure 2A). Of note, the production of IL-12/23p40 was much more pronounced in iCD103 DCs (Figure 2A), while TNF-α was mainly secreted by BMDMs. In addition, BMDMs but not iCD103 DCs produced IL-10 in response to TLR stimulation. Following a similar pattern, at 24 h p.i. *M. bovis* BCG strongly induced the production of IL-12/23p40 by iCD103 DCs, and to a lesser extent of IL-6 and TNF-α. In contrast, production of IL-10 and TNF-α in response to *M. bovis* BCG was more prominent in BMDMs than in iCD103 DCs. Altogether our data suggest that while BMDMs become preferentially infected with *M. bovis* BCG, iCD103 DCs mainly upregulate costimulatory molecules and secrete pro-inflammatory cytokines.

Activation of macrophages and DCs and production of inflammatory cytokines is a crucial step in the induction of adaptive immunity against mycobacterial infection. In addition to Th1 cells, Th17 cells were described to be important for protection against *Mtb*, especially against highly virulent *Mtb* strains and for the effectiveness of mucosal vaccines (52–54). Thus, we tested the capacity of iCD103 DCs and BMDMs to prime and polarize naïve CD4^+^ T cells toward Th1 or Th17 cells. To this aim, iCD103 DCs and BMDMs from WT mice were infected with different MOI of *M. bovis* BCG overexpressing Ag85B (BCG Ag85B) for 24 h and subsequently co-cultured with naïve CD4^+^CD25^−^ P25ktk T cells, expressing a TCR that is specific for peptide P25 of Ag85B bound to I-A^b^ (43). Under non-polarizing conditions, iCD103 DCs induced strong T cell proliferation and IFN-γ production, whereas BMDMs were unable to prime T cells (Figure 2B). Since IL-17 was only marginally induced (data not shown), we also performed co-cultures adding TGF-β and blocking IFN-γ, IL-12/23p40, and IL-4. Under these Th17-skewing conditions iCD103 DCs, but not BMDMs, were able to promote IL-17 production (Figure 2B). These results indicate that iCD103 DCs are highly efficient at priming anti-mycobacterial effector Th1/17 responses.

### iCD103 DCs and BMDMs Upregulate Lipid Synthesis Upon Infection

**Figure 3:**
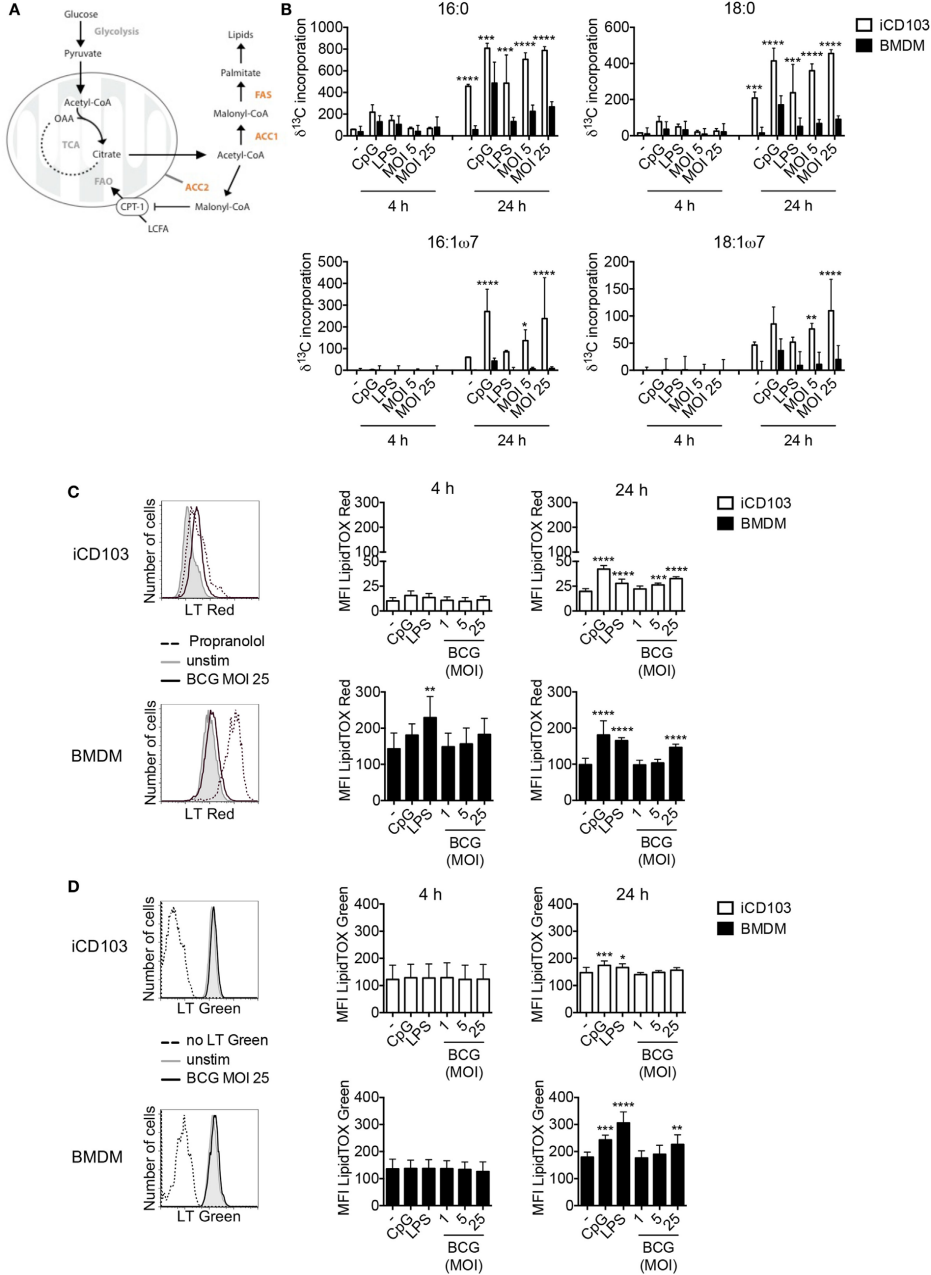
iCD103 dendritic cells (DCs) and macrophages upregulate lipid metabolism upon infection. iCD103 DCs and bone marrow-derived macrophages (BMDMs) were infected with *Mycobacterium bovis* BCG RFP and metabolic parameters were determined at 4 and 24 h p.i. LPS and CpG were used as stimulation controls. **(A)** Schematic overview depicting the glycolytic-lipogenic pathway regulated by acetyl-CoA carboxylase (ACC1) and ACC2. CPT1, carnithine palmitoyl transferase 1; LCFA, long chain fatty acids; FAO, fatty acid oxidation; FAS, fatty acid synthase; OAA, oxaloacetate; TCA, tricarboxylic acid cycle. **(B)** Incorporation (δ) of ^13^C into fatty acids after 4 and 24 h of culture in the presence of [U-^13^C_6_] glucose. **(C,D)** Accumulation of phospholipids **(C)** and neutral lipids **(D)** in iCD103 DCs and BMDMs was assessed by LipidTox (LT) Red **(C)** or LT Green **(D)** staining, respectively. Representative histograms (left panel) depict LT Red/Green staining in unstimulated iCD103 DCs and BMDMs (gray) or cells infected with MOI 25 of *M. bovis* BCG (black) after 24 h. The dashed line represents the positive control Propranolol for LT Red staining **(C)** or an unstained control for LT Green staining **(D)**. Bar graphs show the MFI of LT Red/Green expression (right panel). Results are pooled from three **(B)** or five **(C,D)** experiments. Error bars represent SD of pooled data **(B–D)**. *P < 0.05, **P < 0.01, ***P < 0.001, ****P < 0.0001, one-way ANOVA with Dunnett’s correction **(C,D)**, or two-way ANOVA with Bonferroni correction **(B)**.

Recently it was proposed that upon stimulation, immune cells undergo a metabolic switch from OXPHOS toward aerobic glycolysis, also known as the Warburg effect (55, 56). This metabolic reprogramming observed in macrophages upon infection with *Mtb*, subsequently induces *de novo* cholesterol and FAS, which results in lipid accumulation and so-called “foamy” macrophages (57). In DCs, commitment to aerobic glycolysis upon TLR stimulation was reported to be crucial for activation by supporting *de novo* FAS (30). Thus, we investigated whether DCs and macrophages upregulate the glycolytic-lipogenic pathway upon mycobacterial infection. To this aim, we cultured iCD103 DCs and BMDMs with *M. bovis* BCG or TLR stimuli in the presence of ^13^C-glucose and tracked the incorporation of glucose-derived carbons into lipids (Figure 3A). Both iCD103 DCs and BMDMs increased the incorporation of ^13^C-glucose-derived carbons into saturated fatty acids, such as palmitic acid (C16:0) and stearic acid (C18:0) as well as the unsaturated fatty acids palmitoleic acid (C16:1ω7) and vaccenic acid (C18:1ω7) upon infection (Figure 3B). In general, BMDMs showed lower incorporation of ^13^C-glucose into fatty acids than iCD103 DCs in the basal state, but also upon infection or TLR stimulation (Figure 3B).

Lipids are important components of cellular membranes and they also participate in different signaling events within the cell. For DCs it was proposed that the induction of FAS upon TLR stimulation facilitates DC activation by generating membranes to promote Golgi and ER expansion and increased function (30). In line with this, infection with *M. bovis* BCG induced the accumulation of cellular phospholipids in iCD103 DCs as well as in BMDMs after 24 h, as evaluated by LipidTOX (LT) Red staining (Figure 3C). By confocal microscopy, we observed LT Red stained vesicle-shaped structures (Figure 1B), sometimes co-localizing with BCG, with variable levels of intensity between different cells, regardless of the degree of infection (data not shown). Therefore, in order to have a more quantitative and less biased analysis, we determined the phospholipid and neutral lipid content by flow cytometry. The MFI values for LT Red increased over time and were about 5–10-fold higher in BMDMs than iCD103 DCs, despite greater ^13^C-glucose incorporation into fatty acids in iCD103 DCs (Figure 3B). Moreover, we assessed the accumulation of total neutral lipids by LT Green staining (Figure 3D). While the basal level of neutral lipids was comparable between iCD103 DCs and BMDMs, the accumulation of lipids upon TLR stimulation or *M. bovis* BCG infection at 24 h was more pronounced in BMDMs (Figure 3D). Altogether, these results demonstrate that not only BMDMs, as reported previously, but also DCs upregulate *de novo* FAS and accumulate lipids upon mycobacterial infection.

### ACC1-Mediated *De Novo* FAS is Dispensable for the Function of BMDMs and iCD103 DCs

**Figure 4:**
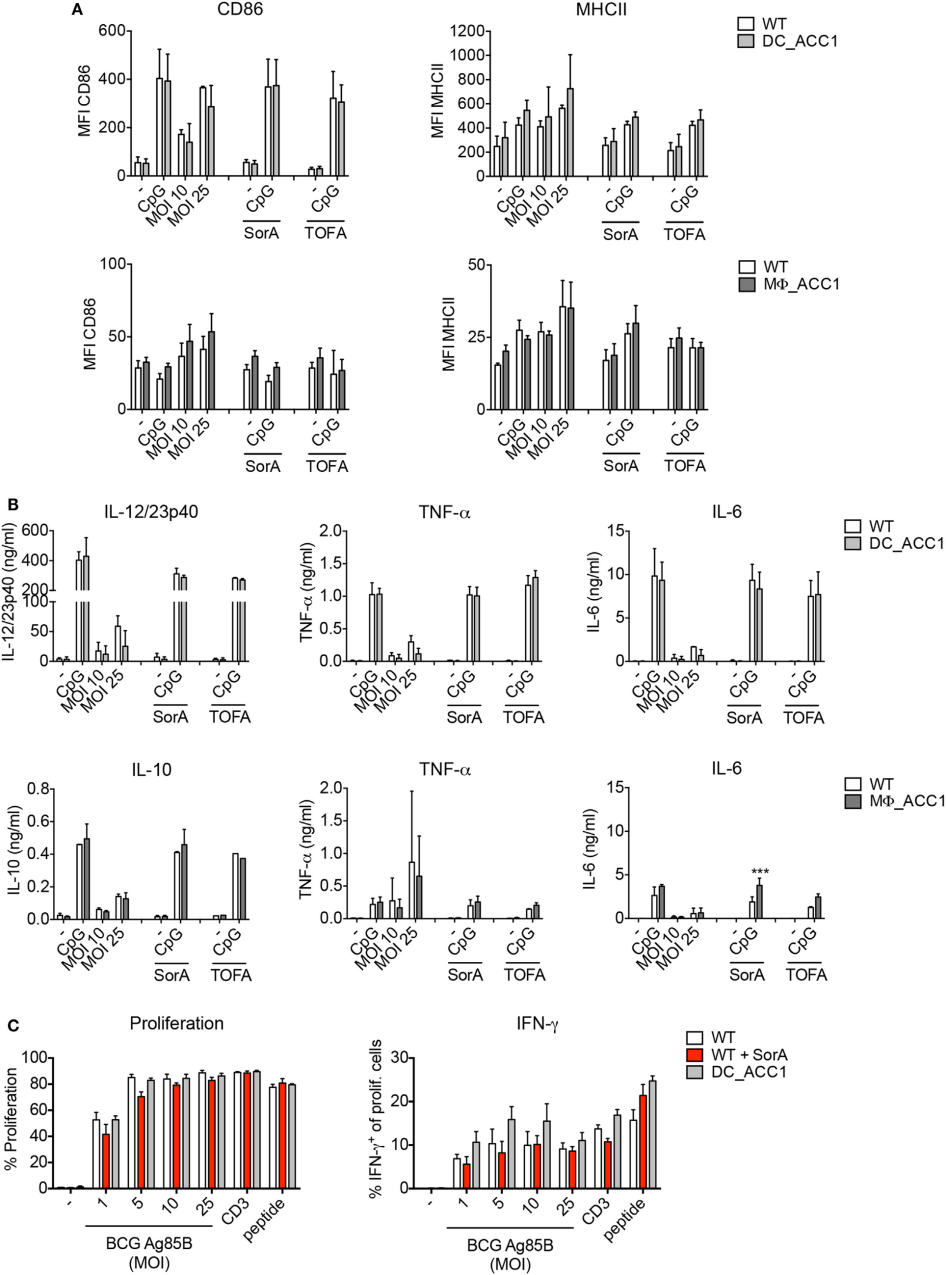
Acetyl-CoA carboxylase (ACC1)-mediated *de novo* fatty acid synthesis (FAS) is dispensable for the function of dendritic cells (DCs) and macrophages. **(A–C)** iCD103 DCs from WT and DC_ACC1 mice or bone marrow-derived macrophages (BMDMs) from WT and MΦ_ACC1 mice were infected with different multiplicities of infections (MOIs) of *Mycobacterium bovis* BCG and *de novo* FAS, activation and function were analyzed 24 h p.i. LPS and CpG served as stimulation controls and SorA and 5-(Tetradecyloxy)-2-furoic acid (TOFA) as inhibitors of ACC1. **(A)** Bar graphs show the MFI of CD86 and MHCII expression determined by flow cytometry. **(B)** Bar graphs display the cytokine levels of IL-12/23p40, TNF-α, IL-6, and IL-10 measured by ELISA. **(C)** iCD103 DCs from WT or DC_ACC1 mice were infected with *M. bovis* BCG overexpressing Ag85B in the presence or absence of SorA. After 24 h, cells were washed and co-cultured with naïve CD4^+^CD25^−^ P25ktk T cells for 4 days. Bar graphs display the proliferation rate and frequencies of IFN-γ^+^ among total live CD4^+^ T cells upon re-stimulation with PMA/ionomycin. Results are pooled from two [**(A)** BMDMs, **(B)** iCD103 DCs] or three [**(A)** iCD103 DCs, **(B)** BMDMs] experiments or shown as a representative of two **(C)** experiments. Each individual experiment had triplicates **(A,B)** or quadruplicates **(C)**. Bar graphs show mean with error bars of SD. *P < 0.05 and **P < 0.01, ***P < 0.001, ****P < 0.0001, n.s., non-significant, two-way ANOVA with Bonferroni correction **(A,B)**.

*De novo* FAS is controlled by the rate of malonyl-CoA production from acetyl-CoA *via* the enzyme ACC1 present in the cytosol. We therefore thought to address whether ACC1 deletion has an impact on DC and macrophage function upon mycobacterial infection. To this aim, we generated DC- and macrophage-specific ACC1-deficient mice (DC_ACC1 and MΦ_ACC1, respectively) by crossing mice carrying a loxP-flanked biotin carboxyl carrier protein domain in the *Acaca* gene (ACC1^*flox/flox*^) (36) with mice expressing the *cre* recombinase under the control of the CD11c (Integrin alpha X) promoter (41) or the lysozyme M (LysM) promoter (40). We then cultured iCD103 DCs and BMDMs from BM of DC_ACC1 and MΦ_ACC1 mice, respectively, and analyzed their activation status and function after 24 h of infection with *M. bovis* BCG. ACC1-deficient iCD103 DCs behaved similarly to their WT counterparts in terms of CD86 and MHCII expression (Figure 4A, upper panel) and pro-inflammatory cytokine production (Figure 4B, upper panel; Figure S1A in Supplementary Material, upper panel). They also showed no defects in their ability to prime P25-specific CD4^+^ T cells (Figure 4C). Likewise, BMDMs from MΦ_ACC1 mice had no defects in their expression of surface molecules (Figure 4A, lower panel) or their production of IL-10, TNF-α, IL-6, and IL-1β compared to WT controls (Figure 4B, lower panel; Figure S1A in Supplementary Material, lower panel).

Pharmacological inhibition of *de novo* FAS impairs the capacity of GM-CSF-derived DCs to produce cytokines and become activated in response to TLR ligation (30). Since we observed no effect of ACC1 deletion in iCD103 DCs, we next tested whether GM-CSF DCs from DC_ACC1 mice become properly activated upon TLR stimulation or infection with *M. bovis* BCG. However, compared to WT cells, we found no differences in the expression of the surface molecules CD86 and MHCII or the levels of IL-12/23p40 and TNF-α, when ACC1-deficient cells were stimulated (Figures S1B,C in Supplementary Material).

Subsequently, we addressed whether the deletion of ACC1 would result in lower rates of FAS. Thus, we evaluated the rate of ^13^C-glucose incorporation into fatty acids. To our surprise, we observed only a slight reduction of *de novo* FAS in ACC1-deficient iCD103 DCs and BMDMs (Figure S2A in Supplementary Material). In order to determine if residual ACC activity was obscuring our results, we assessed the targeting efficiency of the CD11c *cre* and LysM *cre* promoters for ACC1 in these *in vitro*-generated cells. The rate of ACC1 deletion was around 50% for both iCD103 DCs and BMDMs, which could account for the small differences observed in *de novo* FAS rates between WT and transgenic cells (Figure S2B in Supplementary Material). In GM-CSF DCs generated from DC_ACC1 mice, the deletion rate was higher, averaging 70% (Figure S2B in Supplementary Material) and *de novo* FAS from glucose was halved upon ACC1 deletion (data not shown). Additionally, we assessed two pharmacological ACC inhibitors, Soraphen A (SorA) and TOFA in our experiments. Treatment with SorA, a natural compound derived from the myxobacterium *Sorangium cellulosum* (58–60) completely abrogated *de novo* FAS from glucose in iCD103 DCs and BMDMs (Figure S2A in Supplementary Material). However, it had no effect in TLR-driven induction of costimulatory molecules (Figure 4A) or pro-inflammatory cytokines (Figure 4B; Figure S1A in Supplementary Material). TOFA, a potent and widely characterized inhibitor that has been used in several studies (30, 33, 61, 62) also failed to inhibit iCD103 DC and BMDM activation (Figures 4A,B; Figure S1A in Supplementary Material). Moreover, complete ACC inhibition by SorA did not affect the T cell priming capacity of iCD103 DCs (Figure 4C). In conclusion, our results suggest that the inhibition of ACC1-mediated FAS in DCs and macrophages does not compromise their ability to become efficiently activated or to prime effective anti-mycobacterial immune responses *in vitro*.

Interestingly, ^13^C-palmitate and Bodipy-palmitate uptake assays (Figures S3A,B in Supplementary Material) revealed that ACC1 deletion or pharmacological inhibition in BMDMs, and more prominently in iCD103 DCs, results in increased fatty acid uptake. This implies that DCs and macrophages with impaired lipogenesis might exhibit compensatory mechanisms, i.e., augmented uptake of external lipids, to meet their biosynthetic requirements.

### ACC1 Expression in DCs and Macrophages Is Not a Prerequisite for Inflammatory Cytokine Production or T Cell Priming *In Vivo*

**Figure 5:**
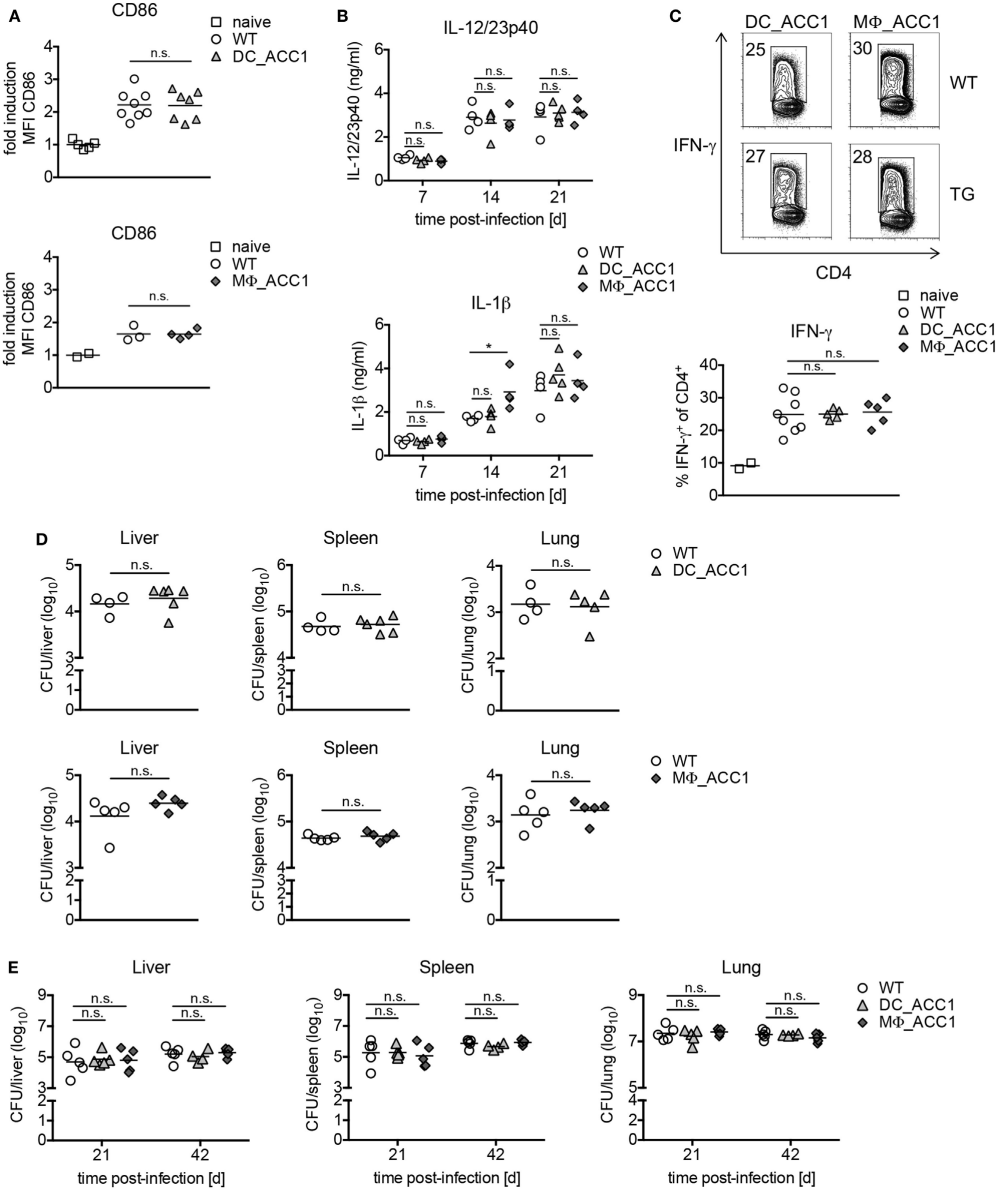
Acetyl-CoA carboxylase (ACC1) expression in dendritic cells (DCs) and macrophages is not a prerequisite for mycobacterial control. **(A–D)** WT, DC_ACC1, and MΦ_ACC1 mice were infected i.v. with 2 × 10^6^ colony forming units (CFU) of *Mycobacterium bovis* BCG and analyzed on day 21 p.i. or also on day 7 and 14 p.i. **(B)**. **(A)** The expression of CD86 was analyzed in splenic CD11c^hi^ MHCII^hi^ DCs or F4/80^+^ macrophages by flow cytometry. Graphs show fold induction of CD86 MFI in infected mice relative to naïve mice. **(B)** Graphs display levels of IL-12/23p40 and IL-1β in the spleen at different time points determined by ELISA. **(C)** Representative flow cytometry plots show the percentage of IFN-γ^+^ cells within live CD4^+^ T cells upon re-stimulation with PMA/ionomycin. Graph represents the frequency of IFN-γ^+^ cells within live splenic CD4^+^ T cells. **(D)** Graphs show bacterial burden in liver, spleen, and lung. **(E)** WT, DC_ACC1, and MΦ_ACC1 mice were infected with a high dose of 1,000 CFU *Mycobacterium tuberculosis* (*Mtb*) *via* the aerosol route and the bacterial burden was determined in liver, spleen, and lung on day 21 and 42 p.i. Each symbol represents an individual mouse. Results are pooled from two experiments [**(A)**, upper panel] with *n* = 1–4 mice per group, from one experiment with *n* = 2–4 mice per group [**(A)**, lower panel] or with *n* = 4–5 mice per group **(E)** or as a representative of four individual experiments with *n* = 4–5 mice per group **(C,D)**. *P < 0.05 and **P < 0.01, ***P < 0.001, ****P < 0.0001, n.s., non-significant, Student’s *t*-test **(A,D)**, one-way ANOVA with Dunnett’s correction **(C,E)** or two-way ANOVA with Bonferroni correction **(B)**.

To confirm the suitability of our mouse models to study the relevance of *de novo* FAS in myeloid cells *in vivo*, we next evaluated the targeting efficiency for ACC1 in *ex vivo*-isolated cells. We found it was over 90% for splenic CD11c^+^ DCs from DC_ACC1 mice (Figure S4A in Supplementary Material), and about 40% for MΦ_ACC1 F4/80^+^ splenic macrophages (Figure S4B in Supplementary Material). Importantly, in MΦ_ACC1 mice, peritoneal and AMs displayed deletion efficiencies between 90 and 100% (Figure S4B in Supplementary Material), thus suggesting that our mouse models are suitable to study the importance of *de novo* FAS in DCs and macrophages in the context of mycobacterial infection *in vivo*. To this aim, we infected DC_ACC1 and MΦ_ACC1 mice with *M. bovis BCG via* the i.v. route and analyzed the expression of CD86 in CD11c^+^ or F4/80^+^ cells in the spleen at day 21 p.i. In both DC_ACC1 and MΦ_ACC1 mice, CD86 was upregulated to the same extent as in their WT counterparts (Figure 5A). Next, we evaluated IL-12/23p40 and IL-1β production in the spleens of DC_ACC1 and MΦ_ACC1 at different time points after infection. Both cytokines increased over time, peaking at 21 days p.i. However, no differences were detected between DC_ACC1, MΦ_ACC1, and WT mice (Figure 5B). In accordance, IFN-γ and IL-12/23p40 levels in the serum were also not impaired upon ACC1 deletion on day 21 p.i. neither in DC_ACC1 nor in MΦ_ACC1 mice (Figure S5A in Supplementary Material). These results indicate that the overall production of inflammatory cytokines was not affected by the absence of ACC1 in myeloid cells. We also evaluated the requirement of *de novo* FAS in DCs and macrophages for priming protective T cell immunity during *M. bovis* BCG infection by analyzing T cell responses in the spleen at day 21 p.i. As shown in Figure 5C, the generation of endogenous IFN-γ-secreting Th1 cells was neither impaired in DC_ACC1 mice, nor in MΦ_ACC1 mice.

Finally, to further confirm that ACC1 expression in DCs is not required for T cell priming during infection, WT and DC_ACC1 mice were infected with 2 × 10^6^ *M. bovis* BCG i.v. and on day 9 p.i. CD4^+^CD45.1^+^ T cells from P25ktk mice labeled with the proliferation dye CellTrace Violet were transferred, according to the experimental scheme shown in Figure S6A in Supplementary Material. After 5 days, the proliferation and cytokine production of transferred cells was analyzed in WT and DC_ACC1 mice. However, transferred P25ktk cells proliferated to the same extent in WT as in DC_ACC1 recipients and produced equal amounts of IFN-γ (Figure S6B in Supplementary Material).

Accordingly, 21 days after systemic *M. bovis* BCG infection, both DC_ACC1 and MΦ_ACC1 mice displayed comparable CFU in liver, spleen, and lungs compared to WT mice (Figure 5D). Moreover, we confirmed these results with another ACC1^*flox/flox*^ mouse strain (*Acaca^flox/flox^)*, in which different exons are targeted for deletion (37). We crossed them with CD11c *cre* and LysM *cre* mice to generate DC_Acaca and MΦ_Acaca mice, which were also capable of controlling *M. bovis* BCG infection like WT mice (Figure S5B in Supplementary Material).

To evaluate if deletion of ACC1 in DCs or macrophages would impair the control of *Mtb* infection, we tested the susceptibility of DC_ACC1 and MΦ_ACC1 mice toward a high dose of *Mtb* aerosol infection. The lack of *de novo* FAS in myeloid cells had no impact on bacterial control as evidenced by the CFU counts in liver, spleen, and lungs at day 21 and 42 after infection, which were comparable to WT mice (Figure 5E; Figure S5C in Supplementary Material).

In addition to ACC1, ACC2 a second isoform of ACC can also convert acetyl-CoA to malonyl-CoA. However, ACC2 is present on the outer mitochondrial membrane and does not contribute to FAS, but instead regulates FAO by producing malonyl-CoA that inhibits the CPT1-dependent transport of long-chain fatty acids into the mitochondria (32). To investigate if ACC2 deletion would impact the outcome of mycobacterial infection, we generated mice with a cell-specific deletion of ACC2 in DCs or macrophages by crossing CD11c or LysM *cre* mice to ACC2^*flox/flox*^ (38) mice and infected them with *M. bovis* BCG. Additionally, we used ACC2 complete knockout mice (42) and infected them with 100 CFU *Mtb via* the aerosol route. Still, the absence of ACC2 in DCs or macrophages did not affect mycobacterial control neither in the *M. bovis* BCG model, nor during *Mtb* infection (Figures S7A,B in Supplementary Material).

### ACC1 Deletion in T Cells Increases Susceptibility Toward *M. bovis* BCG Infection

**Figure 6:**
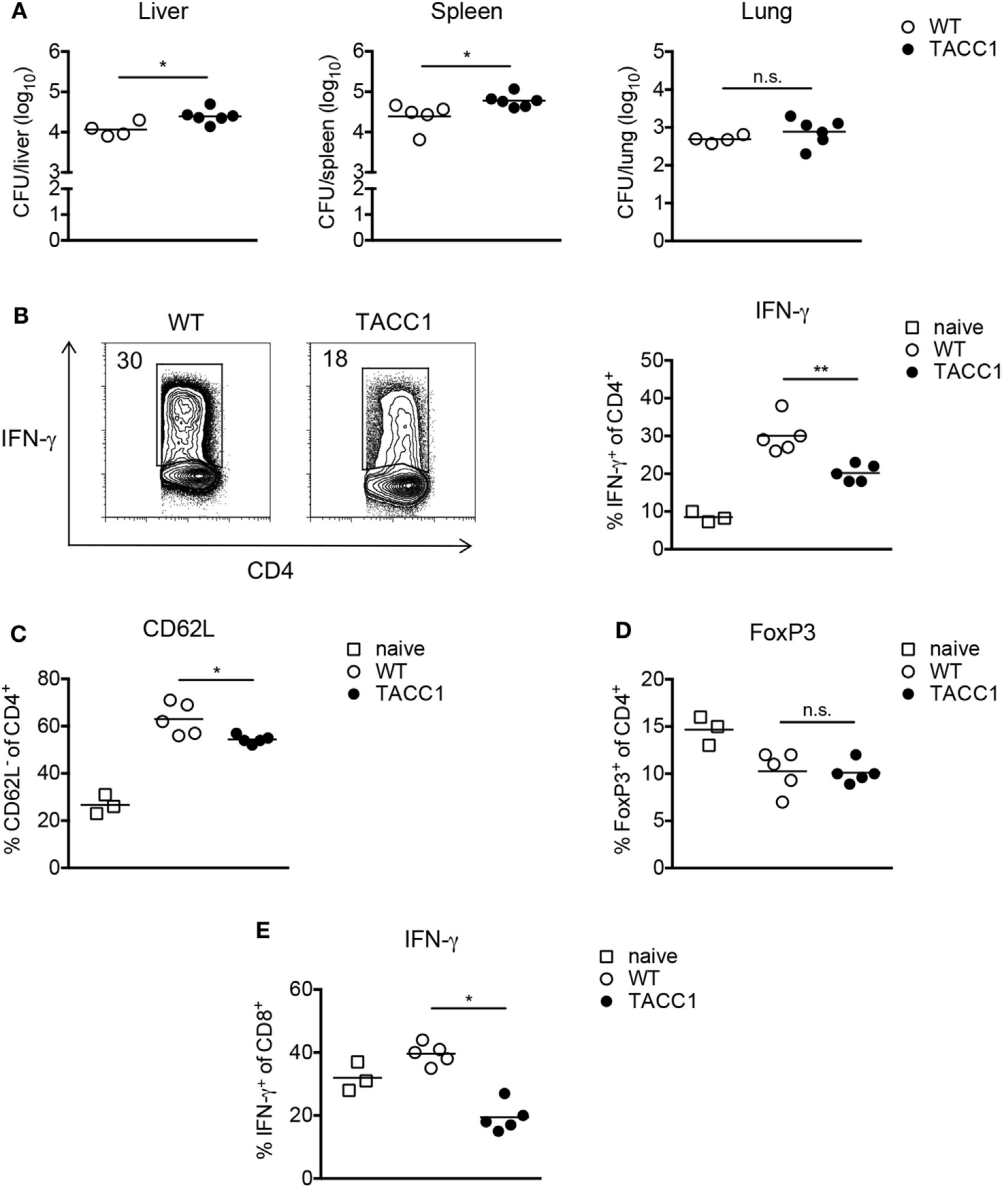
Acetyl-CoA carboxylase (ACC1) deletion in T cells increases susceptibility toward *Mycobacterium bovis* BCG infection. WT and TACC1 mice were infected with 2 × 10^6^ colony forming units (CFU) of *M. bovis* BCG and analyzed on day 21 p.i. **(A)** Bacterial burden in liver, spleen, and lung. **(B–E)** T cell responses were evaluated in spleen 21 days p.i. by flow cytometry. **(B)** Representative flow cytometry plots display the frequency of IFN-γ^+^ cells within live CD4^+^ T cells upon re-stimulation with PMA/ionomycin (left panel). Graph represents the frequency of IFN-γ^+^ cells within live CD4^+^ T cells (right panel). **(C)** Frequency of CD62L^−^ cells within live CD4^+^ T cells. **(D)** Expression of FoxP3 within live CD4^+^ T cells. **(E)** Frequency of IFN-γ^+^ cells within live CD8^+^ T cells. Each symbol represents an individual mouse. Results are representative from three **(C)** or four **(A,B,D,E)** experiments with *n* = 3–6 mice per group. *P < 0.05 and **P < 0.01, ***P < 0.001, ****P < 0.0001, n.s., non-significant, Student’s *t*-test.

Our previous work highlighted the critical dependency of T helper cell differentiation on the glycolytic-lipogenic pathway (33, 35). We therefore speculated that ACC1 deletion in T cells might impair anti-mycobacterial immunity. To test this hypothesis, we infected TACC1 mice that were generated by crossing CD4 *cre* mice to ACC1^*flox/flox*^ mice with *M. bovis* BCG *via* the i.v. route. Indeed, mice lacking ACC1 in T cells exhibited elevated bacterial burden compared to WT littermates (Figure 6A). In TACC1 mice, almost 100% of CD4^+^ T cells carry the targeted deletion (Figure S4C in Supplementary Material). In contrast to ACC1 deficiency, mice with a T cell-specific deletion of ACC2 showed bacterial burden comparable to WT mice (Figure S7C in Supplementary Material).

Analysis of the immune response during infection revealed that T cells from TACC1 mice exhibited reduced frequencies of IFN-γ^+^ CD4 T cells indicating the importance of T cell-intrinsic FAS for mounting Th1 responses (Figure 6B). Additionally, we evaluated the production of IL-17 by CD4^+^ T cells during infection. We did not observe differences in the production of IL-17A among the groups (data not shown). However, the Th17 response in our *M. bovis* BCG infection model was negligible, consisting of only about 0.3–1% IL-17A^+^ cells within the live CD4^+^ T cell compartment. Furthermore, while DC_ACC1 and MΦ_ACC1 mice showed comparable frequencies of CD62L^low^ cells among CD4^+^ T cells compared to WT mice (data not shown), TACC1 displayed lower T cell activation levels (Figure 6C). Of note, in addition to CD4^+^ T cells, also ACC1-deficient CD8^+^ T cells showed a strong reduction in their production of IFN-γ (Figure 6E), suggesting that *de novo* lipid synthesis is also required in these cells to mount effective effector responses during infection.

Inflammatory immune responses are controlled by Tregs, which have been reported to expand in both mice (63) and humans (64–66) upon *Mtb* infection and might serve as a mechanism to establish chronic mycobacterial infection (67). As we have previously shown (45), the frequency of FoxP3^+^ Tregs among live CD4^+^ T cells during *M. bovis* BCG infection is transiently reduced compared to naïve mice (Figure 6D), which can be attributed to the expansion of T effector cells. Given our findings that interfering with *de novo* FAS blocks Th17 and favors Treg development, we speculated that ACC1 deficiency in T cells would lead to an expansion of Tregs during mycobacterial infection, as observed in the experimental autoimmune encephalomyelitis model for human multiple sclerosis (33). However, upon *M. bovis* BCG infection, TACC1 mice, like DC_ACC1 and MΦ_ACC1 mice (data not shown), did not show altered Treg frequencies compared to WT mice (Figure 6D). Taken together, our results highlight the importance of ACC1 expression in T cells to effectively generate an adaptive immunity against *M. bovis* BCG infection.

### ACC1 Expression in T Cells Is Crucial for Immunity Against *Mtb* Infection

**Figure 7:**
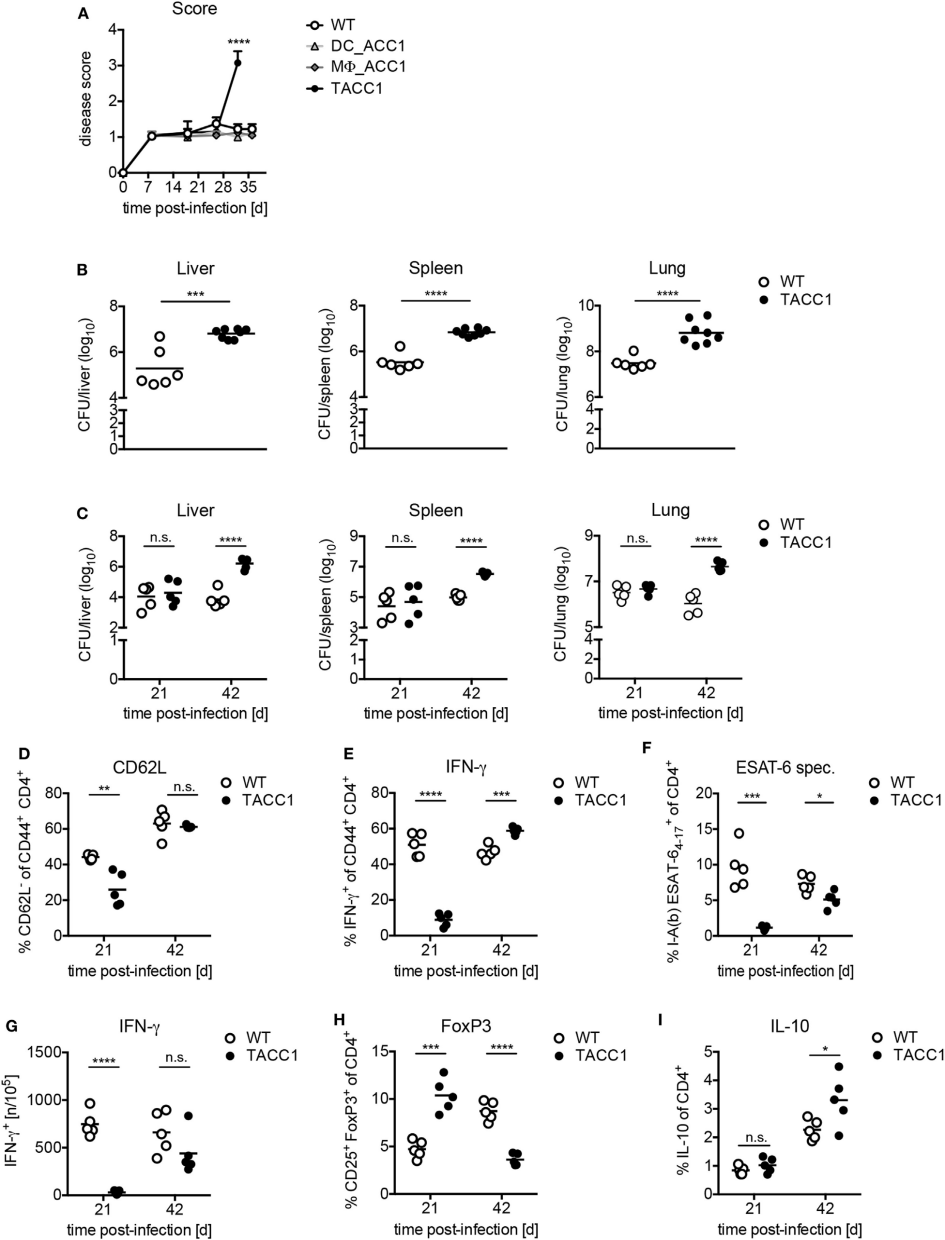
Acetyl-CoA carboxylase (ACC1) expression in T cells is crucial for immunity against *Mycobacterium tuberculosis* (*Mtb*) infection. **(A)** WT, DC_ACC1, MΦ_ACC1, and TACC1 mice were infected with a high dose of 1,000 colony forming units (CFU) *Mtb via* the aerosol route and the disease score was determined during the course of infection. **(B)** WT and TACC1 mice were infected with a high dose of 1,000 CFU *Mtb via* the aerosol route and the bacterial burden was determined in liver, spleen, and lung on day 32 p.i. **(C)** WT and TACC1 mice were infected with a low dose of 100 CFU *Mtb via* the aerosol route and the bacterial burden was determined in liver, spleen, and lung on day 21 and day 42 p.i. **(D–I)** T cell responses were analyzed in the lung of WT and TACC1 mice on day 21 and day 42 p.i. with a low dose of 100 CFU *Mtb via* aerosol route. **(D)** Frequency of CD62L^−^ effector T cells among live CD4^+^CD44^+^ cells. **(E)** Frequency of IFN-γ^+^ T helper 1 cells within live CD4^+^CD44^+^ T cells upon re-stimulation with anti-CD3/CD28. **(F)** Percentage of I-A^b^ *Mtb* ESAT6_4–17_ tetramer^+^ cells among live CD4^+^ T cells. **(G)** Frequency of ESAT6_1–20_-specific IFN-γ^+^ CD4^+^ T cells per 10^5^ total lung cells determined by ELISPOT. **(H)** Frequency of CD25^+^FoxP3^+^ Tregs within live CD4^+^ gate. **(I)** Frequency of IL-10-producing live CD4^+^ T cells. Each symbol represents an individual mouse. Results are from one [**(A)**: DC_ACC1, MΦ_ACC1, **(C–H)**: day 42, **(I)**] experiment or as a representative of two [**(A)** WT, TACC1, **(B,C–H)** day 21] experiments with *n* = 9–10 **(A)** or *n* = 6–8 **(B)** or *n* = 5 **(C–I)** mice per group. *P < 0.05 and **P < 0.01, ***P < 0.001, ****P < 0.0001, n.s., non-significant, Student’s *t*-test.

Finally, to determine the importance of *de novo* FAS in T cells for the control of Tb, we infected mice with a high dose (Figures 7A,B) or low dose aerosol (Figures 7C–I) of *Mtb*. TACC1 mice infected with a high dose reached a high disease score (>3.0) (Figure 7A) and had to be sacrificed at day 32 p.i. At this time point, they exhibited extremely high bacterial burden in all organs tested (Figure 7B). To gain further insights into the immune responses that could explain the susceptibility of TACC1 mice, we made use of the more physiological low dose infection model applying 100 CFU *Mtb via* the aerosol route. Interestingly, while CFU were comparable to WT mice at day 21 p.i., TACC1 mice displayed elevated bacterial burden at 42 days p.i. (Figure 7C). T cell activation, as measured by the downregulation of CD62L expression in CD44^+^CD4^+^ T cells, was reduced early during infection, and restored to WT levels at the later stage (Figure 7D).

Due to the pivotal role of Th1 cells for the protection against *Mtb* (21), we also determined the IFN-γ production by CD4^+^ T cells. On day 21 p.i., we observed that IFN-γ production by CD44^+^CD4^+^ T cells in response to polyclonal CD3/CD28 re-stimulation was drastically impaired, but restored to WT levels at day 42 (Figure 7E). To evaluate whether TACC1 mice were able to generate antigen-specific T cell immunity, we stained lung cells with a tetramer of ESAT-6, a secreted virulence factor and highly immunodominant antigen of *Mtb*. Tetramer staining revealed that TACC1 mice display also a defect in the early generation of ESAT-specific CD4^+^ T cells, which is slightly compensated at day 42, but still reduced compared to WT mice (Figure 7F). In accordance, TACC1 mice also exhibited a delay in the generation of antigen-specific T cells producing IFN-γ, as determined by ELISPOT upon re-stimulation with ESAT6_1–20_-pulsed antigen presenting cells (Figure 7G). Interestingly, the presence of Tregs differed throughout the infection between WT and mice lacking ACC1 in T cells (Figure 7H). After 21 days TACC1 mice showed elevated Treg frequencies compared to WT mice, which was reversed after 42 days. Since Tregs and IL-10 were postulated to limit the host immune response against mycobacteria, we measured the production of IL-10 by T cells in TACC1 during *Mtb* infection. We observed that at day 21 p.i., when Treg frequencies are higher in TACC1 mice, there were no significant differences in T cell-derived IL-10 between WT and transgenic mice (Figure 7I). However, at day 42 p.i., CD4^+^ T cells from TACC1 mice produce more IL-10 than their WT counterparts. Therefore, we cannot definitely exclude that IL-10 dampens the control of mycobacterial growth and contributes to the increased bacterial loads at a later time point of infection. However, as the overall protective T cell response is impaired at earlier time points, we are convinced that this initially compromised reaction accounts for the increased CFUs observed at day 42 p.i. (Figure 7C) independently of Treg-derived IL-10.

Taken together, our data highlight the importance of T cell-intrinsic *de novo* FAS for the generation of protective Th1 immunity against *Mtb* infection. On the contrary, we observed that ACC1 and ACC2 expression in myeloid cells was not essential for the control of mycobacterial infection.

## Discussion

Macrophages and DCs possess distinct roles in the protection against mycobacterial infection. While macrophages control mycobacterial growth using several microbicidal mechanisms, DCs are essential for priming adaptive immunity. In several studies over the past years, these functional properties of immune cells were connected to specific metabolic pathways. Pathogens, such as mycobacteria, have evolved strategies to exploit these metabolic programs for their own survival and replication. In particular, the host cellular lipid metabolism was reported to be modulated by *Mtb*. However, how this metabolic reprogramming affects the function of macrophages and DCs during mycobacterial infection remains unknown. In this study, we demonstrate that in T cells, *de novo* FAS *via* ACC1 is essential to control mycobacterial infection. In contrast, engagement of *de novo* FAS is not a prerequisite for the optimal activation and function of macrophages and DCs.

Previous studies investigating the importance and contrasting roles of macrophages and DCs in generating protective immunity against mycobacteria were mainly performed using BM-derived GM-CSF DCs. GM-CSF DCs have been used in a wide range of studies, since they yield large numbers of cells and represent a useful tool to study certain properties of antigen presenting cells, such as their T cell priming capacity. However, it has recently become apparent that these cultures contain not only DCs, but also monocyte-derived macrophages, termed GM-DCs and GM-Macs, respectively (68). Since GM-CSF DCs represent a heterogeneous mixture of myeloid cells, it is difficult to draw conclusions out of this system on the different roles of DCs and macrophages upon mycobacterial infection. To overcome this, we here made use of a novel protocol developed in our laboratory to generate large numbers of CD103^+^-like DCs (iCD103 DCs) *in vitro* (47). In mice, CD103^+^ DCs are classified as conventional DCs that lack CD8α and express low levels of CD11b (69). They represent a rare population of DCs that survey non-lymphoid tissues and migrate to lymph nodes where they prime adaptive immunity. CD103^+^ DCs were associated with protection against Tb, since they are found in higher numbers in the lungs of *Mtb*-resistant mice than in the susceptible DBA/2 strain (27). Additionally, CD103^+^ DCs can migrate from the lungs to the draining-lymph nodes, where they contribute to the control of infection by initiating T cell responses (50). Here, we show that iCD103 DCs have a stronger upregulation of CD86 and MHCII as well as higher production of IL-12/23p40 and IL-6 in comparison to BMDMs. Consequently, only iCD103 DCs strongly promoted T cell proliferation and differentiation from naïve T cells into Th1 and Th17 cells *in vitro*. These data are in accordance with previous studies using GM-CSF DCs and BMDMs (17, 70) or DCs and macrophages from *M. bovis* BCG-infected mice (71). Additionally, it was described that the ability of DCs to kill mycobacteria is relatively low (17, 18), and, therefore, DCs can transport live bacteria to the lymph nodes (19). For this reason, it has been proposed that *Mtb* might use DCs as a “Trojan horse” to spread within the host and establish a persistent infection (72). Our data shows that iCD103 DCs become infected with mycobacteria, but at a lower rate than macrophages. Importantly, iCD103 DCs are unable to produce NO, in contrast to what was described for GM-CSF DCs (73, 74). NO production is one of the main microbicidal mechanisms required for mycobacterial killing (75). In this respect, iCD103 DCs might represent a good model to study how mycobacteria survive in DCs.

One of the main pathways required for DC activation and production of pro-inflammatory cytokines upon mycobacterial infection is the TLR/MyD88 pathway (28). Recently, it was proposed that TLR-driven activation and cytokine production in DCs is dependent on *de novo* FAS, which serves for the expansion of the ER and Golgi apparatus (30). Immune cells gain fatty acids by the glycolytic-lipogenic pathway, a process by which carbons derived from glucose (and other substrates) are converted to acetyl-CoA, which is then used for the synthesis of fatty acids. Alternatively, fatty acids can be incorporated from the environment using transporters, such as the CD36 receptor or fatty acid binding proteins. Fatty acids can be further subjected to FAO in the mitochondria for the generation of ATP or accumulated in the form of triglycerides within LBs. FAO and FAS are regulated by the enzymes ACC1 and ACC2, both catalyzing the conversion of acetyl-CoA to malonyl-CoA. The malonyl-CoA that is produced by ACC2 functions as an allosteric inhibitor of CPT1, the rate-limiting enzyme for the transport of long chain fatty acids into mitochondria for FAO. In contrast, ACC1 is located in the cytosol and represents the rate-limiting enzyme for *de novo* FAS. In the process of FAS, malonyl-CoA is condensed with acetyl-CoA by the fatty acid synthase (FASN) generating long-chain fatty acids which are further modified into lipids that are used for the synthesis of membranes or posttranslational modifications of proteins (34).

In this study, we demonstrate that iCD103 DCs strongly upregulate *de novo* FAS upon mycobacterial infection as shown by the incorporation of ^13^C-labeled glucose into fatty acids. However, ACC1-deficient iCD103 DCs or GM-CSF DCs displayed normal maturation capacity, as demonstrated by CD86 and MHCII expression, and IL-12/23p40 and TNF-α secretion upon *M. bovis* BCG infection. Furthermore, even the stimulation with TLR agonists, such as LPS or CpG did not reveal any reduction in DC activation or cytokine production upon ACC1 deletion *in vitro*. Likewise, treatment of iCD103 DCs with SorA and TOFA, two different pharmacological inhibitors of ACCs, did not significantly influence iCD103 DC activation, cytokine production, or T cell priming capacity, despite completely blocking ACC-mediated *de novo* FAS. It is also important to mention that the concentrations of the inhibitors used in this study had no effect on cell viability. These results contrast those by Everts *et al*. who reported impaired activation of GM-CSF DCs upon TLR stimulation in the presence of TOFA or C75, an inhibitor of FASN (30). Although the reason for this discrepancy is not clear, it needs to be considered that pharmacological inhibitors often carry the risk of off-target effects [our own unpublished data and (76)]. For example, the previously employed FASN inhibitor C75 was shown to have toxic effects by attenuating cellular mitochondrial function (77). Whether the concentrations of C75 used in the study by Everts *et al*. also affect cell viability was not further addressed. In addition, C75 also promotes FAO by inducing CPT1 activity (78). Hence, it is possible that some of the properties attributed to FAS inhibition in DCs are a consequence of unspecific effects.

*In vivo*, FAS was implicated to be essential for the capacity of DCs to prime CD8^+^ T cell responses (30). In contrast, our results demonstrate that deletion of ACC1 in DCs did not abrogate the cytokine production and maturation capacity of myeloid cells or their ability to prime anti-mycobacterial CD8^+^ or CD4^+^ T cell responses *in vivo*. Consequently, DC_ACC1 mice were able to control the infection with *M. bovis* BCG or *Mtb* to the same extent as WT mice. Likewise, DC_ACC1 mice control *Listeria monocytogenes* infection comparable to WT mice (data not shown). Our results also demonstrate that similar to DCs, macrophages upregulate lipid synthesis upon infection with *M. bovis* BCG *in vitro* as evidenced by incorporation of ^13^C-labeled glucose into lipids and accumulation of phospholipids and neutral lipids. However, upon genetic ablation or pharmacological inhibition of ACC1, BMDMs were still able to upregulate costimulatory molecules and produce pro-inflammatory cytokines to the same extent as WT cells. Additionally, FAS was not required for production of IL-1β, IL-12/23p40, or IFN-γ, generation of protective IFN-γ-secreting CD4^+^ T cells or the subsequent control of *M. bovis* BCG or *Mtb* infection. Together, our data clearly argues against a pivotal role of *de novo* FAS for DC or macrophage activation and function for priming protective immunity against mycobacterial infection.

Of note, the targeting efficiency in splenic DCs and AMs was higher than 90% in our transgenic mouse models, thus excluding the possibility of residual ACC1 expression due to incomplete targeting. Although we did not evaluate the deletion rate in lung CD103^+^ DCs in this study, previous work from our laboratory indicates that CD11c *cre* targets about 80% of this cell subset (79). It is important to consider that this genetic approach also partially targets other cell populations. For example, CD11c *cre* also targets AMs in the lung, while LysM *cre* also targets neutrophils (79, 80), probably also excluding a broader role of FAS in other myeloid cell populations.

*Mycobacterium tuberculosis* survives within macrophages, where it leads to formation of LBs and differentiation into “foamy” macrophages (57). This process has been associated with the induction of *de novo* FAS and cholesterol synthesis in the host macrophage (9, 14). Our findings that bacterial burdens in the lung of WT and MΦ_ACC1 mice are comparable suggest that the *Mtb* strain H37Rv does not strictly depend on FAS within host macrophages. Yet, it needs to be considered that in mice, granulomas do not adequately resemble the fibrocaseous structures containing “foamy” macrophages found in humans (81). Therefore, it might be interesting to address the impact of ACC1 deletion in macrophages on the outcome of anti-mycobacterial immunity and *Mtb* survival in other models, such as in the post-primary tuberculosis model or in IL-13-overexpressing mice, where “foamy” macrophages are highly abundant (11, 82). “Foamy” macrophages have also been observed at later stages during classical aerosol *Mtb* infection.

The usage of FAS or FAO in myeloid cells has been implicated as a metabolic switch regulating immunity and tolerance. While inflammatory “M1” macrophages have been associated with glycolysis, “M2” macrophages were proposed to be committed to FAO and OXPHOS (55, 83, 84). These “M2” macrophages were reported to be immunomodulatory and poorly microbicidal (85, 86) resulting in impaired anti-mycobacterial function (87). This is supported by the finding that *Mtb* induces the production of IL-10 resulting in “M2” polarization and compromised macrophage function (88). In contrast, a recent study reported that *Mtb* induces the microRNAs miR-33 and miR-33* in macrophages, which suppressed autophagy, lysosomal function, and FAO (89). Thereby, *Mtb* facilitates the accumulation of LBs and promotes its survival, indicating that manipulation of FAO in macrophages might affect their anti-mycobacterial function. In line with the current thoughts about macrophages, FAO in DCs is connected to tolerogenicity. This is highlighted by studies showing that resveratrol or vitamin D3 and dexamethasone promote FAO and tolerogenic function of DCs (90–92). Moreover, FAO and CPT1 activity were reported recently to be crucial for activation of plasmacytoid DCs upon virus infection (93). Yet, it remains unclear whether the rate of FAO affects the function of DCs and macrophages to cope with mycobacterial infection. Using cell-specific deletion of ACC2 in DCs or macrophages, we could demonstrate that these mice control mycobacterial infection to the same extent as WT mice. In addition, the paradigm that FAO is required for “M2” polarization was questioned recently (76, 94). In these studies, conditional deletion of CPT2 in macrophages, which functions together with CPT1 to transport long-chain fatty acids into the mitochondria for FAO, blocked β-oxidation of fatty acids, yet it did not affect “M2” polarization (76, 94). Furthermore, this study suggests that the previously observed block of “M2” polarization by using etomoxir as a pharmacological inhibitor for CPT1 (84, 95) might be due to off-target effects [our own unpublished data and (76)].

Metabolic reprogramming upon activation was not only associated with the activation and function of myeloid cells, but also shown to be crucial for the proliferation, differentiation, and function of T cells (34, 96, 97). Our previous work showed that the development of T helper cells requires *de novo* FAS (33). ACC1-deficient T cells (TACC1) are less pathogenic than WT T cells during autoimmune encephalomyelitis and in a lethal model of acute GVHD, where they permitted survival of recipient mice (35). Our current results indicate that ACC1 deletion diminished the generation of Th1 and CD8^+^ T cell responses resulting in higher susceptibility against mycobacterial infection. Indeed, when infected with a high aerosol dose of *Mtb*, TACC1 mice succumb to infection as early as 4 weeks, highlighting the importance of *de novo* FAS for IFN-γ production by Th1 and CD8^+^ T cells. These data are in line with previous publications that established Th1 cells as a prerequisite for the defense against mycobacteria, since mice deficient for Th1-inducing cytokines, as IL-12p40 or IFN-γ, succumb to *Mtb* infection due to high bacterial loads (98–101).

Inflammatory responses are controlled by patrolling Tregs that balance anti-mycobacterial immunity and pathology. Studies in human and mice showed that Tregs expand during *Mtb* infection which was associated with higher bacterial burdens and active Tb (63–65). Our recent data indicate that the deletion of ACC1 in T cells enhances iTreg differentiation [(33) and unpublished data]. Therefore, we speculated that elevated levels of Tregs upon ACC1 deletion might contribute to impaired mycobacterial control in TACC1 mice. Yet, we observed normal Treg frequencies upon *M. bovis* BCG infection and only an early increase during *Mtb* infection that was reversed at day 42. Thus, our results suggest that the lack of protection in TACC1 mice can preferentially be attributed to the impaired ability to generate Th1 cells and not to an increase in Treg development.

Interestingly, genetic ablation or pharmacological inhibition of ACC1 in DCs and macrophages results in higher fatty acid uptake. These results suggest that non-proliferating macrophages and DCs are able to compensate for impaired endogenous FAS by increasing the uptake of external fatty acids. We believe these findings support the notion that myeloid cells are flexible in their choice of substrate, being able to shape their metabolic pathways in order to meet their energetic and biosynthetic demands. Despite displaying a similar compensatory mechanism (data not shown), T cells from TACC1 mice show a strong functional defect upon mycobacterial infection. Together, our data suggest that intrinsic ACC1 expression in DCs and macrophages is dispensable for their activation and function to generate protective immunity against mycobacterial infection. In contrast, ACC1 in highly proliferative T cells constitutes a prerequisite to ensure mycobacterial control.

## Ethics Statement

All animal experiments were performed in compliance with the German animal protection law (TierSchG BGBl. I S. 1105; 25.05.1998). The mice were housed and handled in accordance with good animal practice as defined by FELASA and the national animal welfare body GV-SOLAS. All animal experiments were approved by the Lower Saxony Committee on the Ethics of Animal Experiments as well as the responsible state office (Lower Saxony State Office of Consumer Protection and Food Safety) under the permit numbers 33.19-42502-04-17/2472 and 33.9-42502-04-12/0732 or the Animal Research Ethics Board of the Ministry of the Environment [Kiel, Germany—Permit number: V244-30074/2015 (46-4/15)] considering the German Animal Welfare Act.

## Author Contributions

Conceptualization: LB, TS. Investigation: PS, LM, HE, CA-S, MS, MG, FK, AH, PG, and JB. Writing and Visualization: PS, LM, CH, TS, and LB. Supervision and Project Administration: LB, TS, W-RA, and CH. Funding Acquisition: LB, TS.

## Conflict of Interest Statement

The authors declare that the research was conducted in the absence of any commercial or financial relationships that could be construed as a potential conflict of interest.

## Abbreviations

*Mtb, Mycobacterium tuberculosis*; BMDMs, bone marrow-derived macrophages; GM-CSF, granulocyte-macrophage colony-stimulating factor; Itgax, integrin alpha X; LysM, lysozyme M; p.i., post infection; FAS, fatty acid synthesis; FAO, fatty acid oxidation; ACC, acetyl-CoA carboxylase; CPT1, carnitine palmitoyltransferase 1.

## References

[1] Weiss G Schaible UE. Macrophage defense mechanisms against intracellular bacteria. Immunol Rev (2015) 264(1):182–203. 10.1111/imr.12266 25703560 PMC4368383

[2] Deretic V Delgado M Vergne I Master S De Haro S Ponpuak M Autophagy in immunity against *Mycobacterium tuberculosis*: a model system to dissect immunological roles of autophagy. Curr Top Microbiol Immunol (2009) 335:169–88. 10.1007/978-3-642-00302-8_8 19802565 PMC2788935

[3] Xu G Wang J Gaoand GF Liu CH. Insights into battles between *Mycobacterium tuberculosis* and macrophages. Protein Cell (2014) 5(10):728–36. 10.1007/s13238-014-0077-5 24938416 PMC4180456

[4] Shi L Salamon H Eugenin EA Pine R Cooperand A Gennaro ML. Infection with *Mycobacterium tuberculosis* induces the Warburg effect in mouse lungs. Sci Rep (2015) 5:18176. 10.1038/srep18176 26658723 PMC4674750

[5] Subbian S Tsenova L Kim MJ Wainwright HC Visser A Bandyopadhyay N Lesion-specific immune response in granulomas of patients with pulmonary tuberculosis: a pilot study. PLoS One (2015) 10(7):e0132249. 10.1371/journal.pone.0132249 26133981 PMC4489805

[6] Rodriguez-Prados JC Traves PG Cuenca J Rico D Aragones J Martin-Sanz P Substrate fate in activated macrophages: a comparison between innate, classic, and alternative activation. J Immunol (2010) 185(1):605–14. 10.4049/jimmunol.0901698 20498354

[7] Galvan-Pena S O’Neill LA. Metabolic reprograming in macrophage polarization. Front Immunol (2014) 5:420. 10.3389/fimmu.2014.00420 25228902 PMC4151090

[8] Koo MS Subbianand S Kaplan G. Strain specific transcriptional response in *Mycobacterium tuberculosis* infected macrophages. Cell Commun Signal (2012) 10(1):2. 10.1186/1478-811X-10-2 22280836 PMC3317440

[9] Mehrotra P Jamwal SV Saquib N Sinha N Siddiqui Z Manivel V Pathogenicity of *Mycobacterium tuberculosis* is expressed by regulating metabolic thresholds of the host macrophage. PLoS Pathog (2014) 10(7):e1004265. 10.1371/journal.ppat.1004265 25058590 PMC4110042

[10] Gleeson LE Sheedy FJ Palsson-McDermott EM Triglia D O’Leary SM O’Sullivan MP Cutting edge: *Mycobacterium tuberculosis* induces aerobic glycolysis in human alveolar macrophages that is required for control of intracellular bacillary replication. J Immunol (2016) 196(6):2444–9. 10.4049/jimmunol.1501612 26873991

[11] Hunter RL Jagannathand C Actor JK. Pathology of postprimary tuberculosis in humans and mice: contradiction of long-held beliefs. Tuberculosis (Edinb) (2007) 87(4):267–78. 10.1016/j.tube.2006.11.003 17369095

[12] Peyron P Vaubourgeix J Poquet Y Levillain F Botanch C Bardou F Foamy macrophages from tuberculous patients’ granulomas constitute a nutrient-rich reservoir for *M. tuberculosis* persistence. PLoS Pathog (2008) 4(11):e1000204. 10.1371/journal.ppat.1000204 19002241 PMC2575403

[13] Gutierrez MG Master SS Singh SB Taylor GA Colomboand MI Deretic V. Autophagy is a defense mechanism inhibiting BCG and *Mycobacterium tuberculosis* survival in infected macrophages. Cell (2004) 119(6):753–66. 10.1016/j.cell.2004.11.038 15607973

[14] Singh V Jamwal S Jain R Verma P Gokhaleand R Rao KV. *Mycobacterium tuberculosis*-driven targeted recalibration of macrophage lipid homeostasis promotes the foamy phenotype. Cell Host Microbe (2012) 12(5):669–81. 10.1016/j.chom.2012.09.012 23159056

[15] Singh V Kaur C Chaudhary VK Raoand KV Chatterjee S. *M. tuberculosis* secretory protein ESAT-6 induces metabolic flux perturbations to drive foamy macrophage differentiation. Sci Rep (2015) 5:12906. 10.1038/srep12906 26250836 PMC5388048

[16] D’Avila H Melo RC Parreira GG Werneck-Barroso E Castro-Faria-Netoand HC Bozza PT *Mycobacterium bovis* bacillus Calmette-Guerin induces TLR2-mediated formation of lipid bodies: intracellular domains for eicosanoid synthesis in vivo. J Immunol (2006) 176(5):3087–97. 10.4049/jimmunol.176.5.3087 16493068

[17] Bodnar KA Serbinaand NV Flynn JL. Fate of *Mycobacterium tuberculosis* within murine dendritic cells. Infect Immun (2001) 69(2):800–9. 10.1128/IAI.69.2.800-809.2001 11159971 PMC97955

[18] Buettner M Meinken C Bastian M Bhat R Stossel E Faller G Inverse correlation of maturity and antibacterial activity in human dendritic cells. J Immunol (2005) 174(7):4203–9. 10.4049/jimmunol.174.7.4203 15778382

[19] Wolf AJ Linas B Trevejo-Nunez GJ Kincaid E Tamura T Takatsu K *Mycobacterium tuberculosis* infects dendritic cells with high frequency and impairs their function in vivo. J Immunol (2007) 179(4):2509–19. 10.4049/jimmunol.179.4.2509 17675513

[20] Wolf AJ Desvignes L Linas B Banaiee N Tamura T Takatsu K Initiation of the adaptive immune response to *Mycobacterium tuberculosis* depends on antigen production in the local lymph node, not the lungs. J Exp Med (2008) 205(1):105–15. 10.1084/jem.20071367 18158321 PMC2234384

[21] Green AM Difazioand R Flynn JL IFN-gamma from CD4 T cells is essential for host survival and enhances CD8 T cell function during *Mycobacterium tuberculosis* infection. J Immunol (2013) 190(1):270–7. 10.4049/jimmunol.1200061 23233724 PMC3683563

[22] Tian T Woodworth J Skoldand M Behar SM. In vivo depletion of CD11c+ cells delays the CD4+ T cell response to *Mycobacterium tuberculosis* and exacerbates the outcome of infection. J Immunol (2005) 175(5):3268–72. 10.4049/jimmunol.175.5.3268 16116218

[23] Maldonado MA MacDonald GC Kakkanaiah VN Fecho K Dransfield M Sekiguchi D Differential control of autoantibodies and lymphoproliferation by Fas ligand expression on CD4+ and CD8+ T cells in vivo. J Immunol (1999) 163(6):3138–42. 10477580

[24] Hochrein H Shortman K Vremec D Scott B Hertzogand P O’Keeffe M. Differential production of IL-12, IFN-alpha, and IFN-gamma by mouse dendritic cell subsets. J Immunol (2001) 166(9):5448–55. 10.4049/jimmunol.166.9.5448 11313382

[25] Shortman K Heath WR. The CD8+ dendritic cell subset. Immunol Rev (2010) 234(1):18–31. 10.1111/j.0105-2896.2009.00870.x 20193009

[26] Sung SS Fu SM Rose CE Jr Gaskin F Juand ST Beaty SR. A major lung CD103 (alphaE)-beta7 integrin-positive epithelial dendritic cell population expressing Langerin and tight junction proteins. J Immunol (2006) 176(4):2161–72. 10.4049/jimmunol.176.4.2161 16455972

[27] Leepiyasakulchai C Ignatowicz L Pawlowski A Kalleniusand G Skold M. Failure to recruit anti-inflammatory CD103+ dendritic cells and a diminished CD4+ Foxp3+ regulatory T cell pool in mice that display excessive lung inflammation and increased susceptibility to *Mycobacterium tuberculosis*. Infect Immun (2012) 80(3):1128–39. 10.1128/IAI.05552-11 22215739 PMC3294659

[28] Berod L Stuve P Swallow M Arnold-Schrauf C Kruse F Gentilini MV MyD88 signalling in myeloid cells is sufficient to prevent chronic mycobacterial infection. Eur J Immunol (2014) 44(5):1399–409. 10.1002/eji.201344039 24435955

[29] Krawczyk CM Holowka T Sun J Blagih J Amiel E DeBerardinis RJ Toll-like receptor-induced changes in glycolytic metabolism regulate dendritic cell activation. Blood (2010) 115(23):4742–9. 10.1182/blood-2009-10-249540 20351312 PMC2890190

[30] Everts B Amiel E Huang SC Smith AM Chang CH Lam WY TLR-driven early glycolytic reprogramming via the kinases TBK1-IKKvarepsilon supports the anabolic demands of dendritic cell activation. Nat Immunol (2014) 15(4):323–32. 10.1038/ni.2833 24562310 PMC4358322

[31] Herber DL Cao W Nefedova Y Novitskiy SV Nagaraj S Tyurin VA Lipid accumulation and dendritic cell dysfunction in cancer. Nat Med (2010) 16(8):880–6. 10.1038/nm.2172 20622859 PMC2917488

[32] Wakil SJ Abu-Elheiga LA. Fatty acid metabolism: target for metabolic syndrome. J Lipid Res (2009) 50(Suppl):S138–43. 10.1194/jlr.R800079-JLR200 19047759 PMC2674721

[33] Berod L Friedrich C Nandan A Freitag J Hagemann S Harmrolfs K De novo fatty acid synthesis controls the fate between regulatory T and T helper 17 cells. Nat Med (2014) 20(11):1327–33. 10.1038/nm.3704 25282359

[34] Lochner M Berodand L Sparwasser T. Fatty acid metabolism in the regulation of T cell function. Trends Immunol (2015) 36(2):81–91. 10.1016/j.it.2014.12.005 25592731

[35] Raha S Raud B Oberdorfer L Castro CN Schreder A Freitag J Disruption of de novo fatty acid synthesis via acetyl-CoA carboxylase 1 inhibition prevents acute graft-versus-host disease. Eur J Immunol (2016) 46(9):2233–8. 10.1002/eji.201546152 27338930

[36] Mao J DeMayo FJ Li H Abu-Elheiga L Gu Z Shaikenov TE Liver-specific deletion of acetyl-CoA carboxylase 1 reduces hepatic triglyceride accumulation without affecting glucose homeostasis. Proc Natl Acad Sci U S A (2006) 103(22):8552–7. 10.1073/pnas.0603115103 16717184 PMC1570106

[37] Lee J Walsh MC Hoehn KL James DE Wherryand EJ Choi Y. Regulator of fatty acid metabolism, acetyl coenzyme a carboxylase 1, controls T cell immunity. J Immunol (2014) 192(7):3190–9. 10.4049/jimmunol.1302985 24567531 PMC3965631

[38] Olson DP Pulinilkunnil T Cline GW Shulmanand GI Lowell BB. Gene knockout of Acc2 has little effect on body weight, fat mass, or food intake. Proc Natl Acad Sci U S A (2010) 107(16):7598–603. 10.1073/pnas.0913492107 20368432 PMC2867727

[39] Lee PP Fitzpatrick DR Beard C Jessup HK Lehar S Makar KW A critical role for Dnmt1 and DNA methylation in T cell development, function, and survival. Immunity (2001) 15(5):763–74. 10.1016/S1074-7613(01)00227-8 11728338

[40] Clausen BE Burkhardt C Reith W Renkawitzand R Forster I. Conditional gene targeting in macrophages and granulocytes using LysMcre mice. Transgenic Res (1999) 8(4):265–77. 10.1023/A:1008942828960 10621974

[41] Caton ML Smith-Raskaand MR Reizis B. Notch-RBP-J signaling controls the homeostasis of CD8- dendritic cells in the spleen. J Exp Med (2007) 204(7):1653–64. 10.1084/jem.20062648 17591855 PMC2118632

[42] Abu-Elheiga L Matzuk MM Abo-Hashemaand KA Wakil SJ. Continuous fatty acid oxidation and reduced fat storage in mice lacking acetyl-CoA carboxylase 2. Science (2001) 291(5513):2613–6. 10.1126/science.1056843 11283375

[43] Tamura T Ariga H Kinashi T Uehara S Kikuchi T Nakada M The role of antigenic peptide in CD4+ T helper phenotype development in a T cell receptor transgenic model. Int Immunol (2004) 16(12):1691–9. 10.1093/intimm/dxh170 15477229

[44] Schaefer M Reiling N Fessler C Stephani J Taniuchi I Hatam F Decreased pathology and prolonged survival of human DC-SIGN transgenic mice during mycobacterial infection. J Immunol (2008) 180(10):6836–45. 10.4049/jimmunol.180.10.6836 18453604

[45] Berod L Stuve P Varela F Behrends J Swallow M Kruse F Rapid rebound of the Treg compartment in DEREG mice limits the impact of Treg depletion on mycobacterial burden, but prevents autoimmunity. PLoS One (2014) 9(7):e102804. 10.1371/journal.pone.0102804 25050936 PMC4106855

[46] Morton DB Griffiths PH. Guidelines on the recognition of pain, distress and discomfort in experimental animals and an hypothesis for assessment. Vet Rec (1985) 116(16):431–6. 10.1136/vr.116.16.431 3923690

[47] Mayer CT Ghorbani P Nandan A Dudek M Arnold-Schrauf C Hesse C Selective and efficient generation of functional Batf3-dependent CD103+ dendritic cells from mouse bone marrow. Blood (2014) 124(20):3081–91. 10.1182/blood-2013-12-545772 25100743 PMC4260363

[48] Zal T Volkmannand A Stockinger B. Mechanisms of tolerance induction in major histocompatibility complex class II-restricted T cells specific for a blood-borne self-antigen. J Exp Med (1994) 180(6):2089–99. 10.1084/jem.180.6.2089 7964486 PMC2191800

[49] Abraham WR Hesse C. Isotope fractionations in the biosynthesis of cell components by different fungi: a basis for environmental carbon flux studies. FEMS Microbiol Ecol (2003) 46(1):121–8. 10.1016/S0168-6496(03)00203-4 19719589

[50] Koh VH Ng SL Ang ML Lin W Ruedland C Alonso S. Role and contribution of pulmonary CD103+ dendritic cells in the adaptive immune response to *Mycobacterium tuberculosis*. Tuberculosis (Edinb) (2017) 102:34–46. 10.1016/j.tube.2016.12.003 28061951

[51] von Meyenn FM Schaefer H Weighardt S Bauer C Kirschning J Wagner H Toll-like receptor 9 contributes to recognition of *Mycobacterium bovis* bacillus Calmette-Guerin by Flt3-ligand generated dendritic cells. Immunobiology (2006) 211(6–8):557–65. 10.1016/j.imbio.2006.05.004 16920494

[52] Khader SA Bell GK Pearl JE Fountain JJ Rangel-Moreno J Cilley GE IL-23 and IL-17 in the establishment of protective pulmonary CD4+ T cell responses after vaccination and during *Mycobacterium tuberculosis* challenge. Nat Immunol (2007) 8(4):369–77. 10.1038/ni1449 17351619

[53] Gopal R Rangel-Moreno J Slight S Lin Y Nawar HF Fallert Junecko BA Interleukin-17-dependent CXCL13 mediates mucosal vaccine-induced immunity against tuberculosis. Mucosal Immunol (2013) 6(5):972–84. 10.1038/mi.2012.135 23299616 PMC3732523

[54] Gopal R Monin L Slight S Uche U Blanchard E Fallert Junecko BA Unexpected role for IL-17 in protective immunity against hypervirulent *Mycobacterium tuberculosis* HN878 infection. PLoS Pathog (2014) 10(5):e1004099. 10.1371/journal.ppat.1004099 24831696 PMC4022785

[55] Pearce EL Pearce EJ. Metabolic pathways in immune cell activation and quiescence. Immunity (2013) 38(4):633–43. 10.1016/j.immuni.2013.04.005 23601682 PMC3654249

[56] Ganeshan K Chawla A. Metabolic regulation of immune responses. Annu Rev Immunol (2014) 32:609–34. 10.1146/annurev-immunol-032713-120236 24655299 PMC5800786

[57] Russell DG Cardona PJ Kim MJ Allainand S Altare F. Foamy macrophages and the progression of the human tuberculosis granuloma. Nat Immunol (2009) 10(9):943–8. 10.1038/ni.1781 19692995 PMC2759071

[58] Gerth K Bedorf N Irschik H Hofleand G Reichenbach H. The soraphens: a family of novel antifungal compounds from *Sorangium cellulosum* (Myxobacteria). I. Soraphen A1 alpha: fermentation, isolation, biological properties. J Antibiot (Tokyo) (1994) 47(1):23–31. 10.7164/antibiotics.47.23 8119858

[59] Vahlensieck HF Pridzun L Reichenbachand H Hinnen A. Identification of the yeast ACC1 gene product (acetyl-CoA carboxylase) as the target of the polyketide fungicide soraphen A. Curr Genet (1994) 25(2):95–100. 10.1007/BF00309532 7916271

[60] Shen Y Volrath SL Weatherly SC Elichand TD Tong L. A mechanism for the potent inhibition of eukaryotic acetyl-coenzyme A carboxylase by soraphen A, a macrocyclic polyketide natural product. Mol Cell (2004) 16(6):881–91. 10.1016/j.molcel.2004.11.034 15610732

[61] Endo Y Asou HK Matsugae N Hirahara K Shinoda K Tumes DJ Obesity drives Th17 cell differentiation by inducing the lipid metabolic kinase, ACC1. Cell Rep (2015) 12(6):1042–55. 10.1016/j.celrep.2015.07.014 26235623

[62] Angela M Endo Y Asou HK Yamamoto T Tumes DJ Tokuyama H Fatty acid metabolic reprogramming via mTOR-mediated inductions of PPARgamma directs early activation of T cells. Nat Commun (2016) 7:13683 10.1038/ncomms13683 27901044 PMC5141517

[63] Scott-Browne JP Shafiani S Tucker-Heard G Ishida-Tsubota K Fontenot JD Rudensky AY Expansion and function of Foxp3-expressing T regulatory cells during tuberculosis. J Exp Med (2007) 204(9):2159–69. 10.1084/jem.20062105 17709423 PMC2118702

[64] Guyot-Revol V Innes JA Hackforth S Hinksand T Lalvani A. Regulatory T cells are expanded in blood and disease sites in patients with tuberculosis. Am J Respir Crit Care Med (2006) 173(7):803–10. 10.1164/rccm.200508-1294OC 16339919

[65] Chen X Zhou B Li M Deng Q Wu X Le X CD4(+)CD25(+)FoxP3(+) regulatory T cells suppress *Mycobacterium tuberculosis* immunity in patients with active disease. Clin Immunol (2007) 123(1):50–9. 10.1016/j.clim.2006.11.009 17234458

[66] Hougardy JM Verscheure V Lochtand C Mascart F. In vitro expansion of CD4+CD25highFOXP3+CD127low/- regulatory T cells from peripheral blood lymphocytes of healthy *Mycobacterium tuberculosis*-infected humans. Microbes Infect (2007) 9(11):1325–32. 10.1016/j.micinf.2007.06.004 17890131

[67] Berod L Puttur F Huehnand J Sparwasser T. Tregs in infection and vaccinology: heroes or traitors? Microb Biotechnol (2012) 5(2):260–9. 10.1111/j.1751-7915.2011.00299.x 21951341 PMC3815786

[68] Helft J Bottcher J Chakravarty P Zelenay S Huotari J Schraml BU GM-CSF mouse bone marrow cultures comprise a heterogeneous population of CD11c(+)MHCII(+) macrophages and dendritic cells. Immunity (2015) 42(6):1197–211. 10.1016/j.immuni.2015.05.018 26084029

[69] Ginhoux F Liu K Helft J Bogunovic M Greter M Hashimoto D The origin and development of nonlymphoid tissue CD103+ DCs. J Exp Med (2009) 206(13):3115–30. 10.1084/jem.20091756 20008528 PMC2806447

[70] Grace PS Ernst JD. Suboptimal antigen presentation contributes to virulence of *Mycobacterium tuberculosis* in vivo. J Immunol (2016) 196(1):357–64. 10.4049/jimmunol.1501494 26573837 PMC4684992

[71] Jiao X Lo-Man R Guermonprez P Fiette L Deriaud E Burgaud S Dendritic cells are host cells for mycobacteria in vivo that trigger innate and acquired immunity. J Immunol (2002) 168(3):1294–301. 10.4049/jimmunol.168.3.1294 11801668

[72] Herrmann JL Lagrange PH. Dendritic cells and *Mycobacterium tuberculosis*: which is the Trojan horse? Pathol Biol (Paris) (2005) 53(1):35–40. 10.1016/j.patbio.2004.01.004 15620608

[73] Bonham CA Lu L Hoffman RA Simmonsand RL Thomson AW Generation of nitric oxide by mouse dendritic cells and its implications for immune response regulation. Adv Exp Med Biol (1997) 417:283–90. 10.1007/978-1-4757-9966-8_46 9286374

[74] Everts B Amiel E van der Windt GJ Freitas TC Chott R Yarasheski KE Commitment to glycolysis sustains survival of NO-producing inflammatory dendritic cells. Blood (2012) 120(7):1422–31. 10.1182/blood-2012-03-419747 22786879 PMC3423780

[75] Adams LB Dinauer MC Morgensternand DE Krahenbuhl JL. Comparison of the roles of reactive oxygen and nitrogen intermediates in the host response to *Mycobacterium tuberculosis* using transgenic mice. Tuber Lung Dis (1997) 78(5–6):237–46. 10.1016/S0962-8479(97)90004-6 10209678

[76] Nomura M Liu J Rovira II Gonzalez-Hurtado E Lee J Wolfgang MJ Fatty acid oxidation in macrophage polarization. Nat Immunol (2016) 17(3):216–7. 10.1038/ni.3366 26882249 PMC6033271

[77] Chen C Han X Zou X Li Y Yang L Cao K 4-methylene-2-octyl-5-oxotetrahydrofuran-3-carboxylic acid (C75), an inhibitor of fatty-acid synthase, suppresses the mitochondrial fatty acid synthesis pathway and impairs mitochondrial function. J Biol Chem (2014) 289(24):17184–94. 10.1074/jbc.M114.550806 24784139 PMC4059159

[78] Thupari JN Landree LE Ronnettand GV Kuhajda FP. C75 increases peripheral energy utilization and fatty acid oxidation in diet-induced obesity. Proc Natl Acad Sci U S A (2002) 99(14):9498–502. 10.1073/pnas.132128899 12060712 PMC123169

[79] Dudek M Puttur F Arnold-Schrauf C Kuhl AA Holzmann B Henriques-Normark B Lung epithelium and myeloid cells cooperate to clear acute pneumococcal infection. Mucosal Immunol (2016) 9(5):1288–302. 10.1038/mi.2015.128 26627460 PMC4990776

[80] Abram CL Roberge GL Huand Y Lowell CA. Comparative analysis of the efficiency and specificity of myeloid-Cre deleting strains using ROSA-EYFP reporter mice. J Immunol Methods (2014) 408:89–100. 10.1016/j.jim.2014.05.009 24857755 PMC4105345

[81] Russell DG Barry CE III Flynn JL. Tuberculosis: what we don’t know can, and does, hurt us. Science (2010) 328(5980):852–6. 10.1126/science.1184784 20466922 PMC2872107

[82] Heitmann L Abad Dar M Schreiber T Erdmann H Behrends J McKenzie AN The IL-13/IL-4Ralpha axis is involved in tuberculosis-associated pathology. J Pathol (2014) 234(3):338–50. 10.1002/path.4399 24979482 PMC4277691

[83] Odegaard JI Chawla A. The immune system as a sensor of the metabolic state. Immunity (2013) 38(4):644–54. 10.1016/j.immuni.2013.04.001 23601683 PMC3663597

[84] Huang SC Everts B Ivanova Y O’Sullivan D Nascimento M Smith AM Cell-intrinsic lysosomal lipolysis is essential for alternative activation of macrophages. Nat Immunol (2014) 15(9):846–55. 10.1038/ni.2956 25086775 PMC4139419

[85] Raju B Hoshino Y Belitskaya-Lévy I Dawson R Ress S Gold JA Gene expression profiles of bronchoalveolar cells in pulmonary TB. Tuberculosis (Edinb) (2008) 88(1):39–51. 10.1016/j.tube.2007.07.003 17921069 PMC3151146

[86] Martinez FO Helmingand L Gordon S. Alternative activation of macrophages: an immunologic functional perspective. Annu Rev Immunol (2009) 27:451–83. 10.1146/annurev.immunol.021908.132532 19105661

[87] Kahnert A Seiler P Stein M Bandermann S Hahnke K Mollenkopf H Alternative activation deprives macrophages of a coordinated defense program to *Mycobacterium tuberculosis*. Eur J Immunol (2006) 36(3):631–47. 10.1002/eji.200535496 16479545

[88] Schreiber T Ehlers S Heitmann L Rausch A Mages J Murray PJ Autocrine IL-10 induces hallmarks of alternative activation in macrophages and suppresses antituberculosis effector mechanisms without compromising T cell immunity. J Immunol (2009) 183(2):1301–12. 10.4049/jimmunol.0803567 19561100 PMC2735238

[89] Ouimet M Koster S Sakowski E Ramkhelawon B van Solingen C Oldebeken S *Mycobacterium tuberculosis* induces the miR-33 locus to reprogram autophagy and host lipid metabolism. Nat Immunol (2016) 17(6):677–86. 10.1038/ni.3434 27089382 PMC4873392

[90] Svajger U Obermajerand N Jeras M. Dendritic cells treated with resveratrol during differentiation from monocytes gain substantial tolerogenic properties upon activation. Immunology (2010) 129(4):525–35. 10.1111/j.1365-2567.2009.03205.x 20002210 PMC2842499

[91] Ferreira GB Vanherwegen AS Eelen G Gutierrez AC Van Lommel L Marchal K Vitamin D3 induces tolerance in human dendritic cells by activation of intracellular metabolic pathways. Cell Rep (2015) 10(5):711–25. 10.1016/j.celrep.2015.01.013 25660022

[92] Malinarich F Duan K Hamid RA Bijin A Lin WX Poidinger M High mitochondrial respiration and glycolytic capacity represent a metabolic phenotype of human tolerogenic dendritic cells. J Immunol (2015) 194(11):5174–86. 10.4049/jimmunol.1303316 25917094

[93] Wu D Sanin DE Everts B Chen Q Qiu J Buck MD Type 1 interferons induce changes in core metabolism that are critical for immune function. Immunity (2016) 44(6):1325–36. 10.1016/j.immuni.2016.06.006 27332732 PMC5695232

[94] Gonzalez-Hurtado E Lee J Choi J Selen Alpergin ES Collins SL Horton MR Loss of macrophage fatty acid oxidation does not potentiate systemic metabolic dysfunction. Am J Physiol Endocrinol Metab (2017) 312(5):E381–93. 10.1152/ajpendo.00408.2016 28223293 PMC5451524

[95] Vats D Mukundan L Odegaard JI Zhang L Smith KL Morel CR Oxidative metabolism and PGC-1beta attenuate macrophage-mediated inflammation. Cell Metab (2006) 4(1):13–24. 10.1016/j.cmet.2006.08.006 16814729 PMC1904486

[96] MacIver NJ Michalekand RD Rathmell JC. Metabolic regulation of T lymphocytes. Annu Rev Immunol (2013) 31:259–83. 10.1146/annurev-immunol-032712-095956 23298210 PMC3606674

[97] Almeida L Lochner M Berodand L Sparwasser T. Metabolic pathways in T cell activation and lineage differentiation. Semin Immunol (2016) 28(5):514–24. 10.1016/j.smim.2016.10.009 27825556

[98] Cooper AM Dalton DK Stewart TA Griffin JP Russelland DG Orme IM. Disseminated tuberculosis in interferon gamma gene-disrupted mice. J Exp Med (1993) 178(6):2243–7. 10.1084/jem.178.6.2243 8245795 PMC2191280

[99] Cooper AM Magram J Ferranteand J Orme IM. Interleukin 12 (IL-12) is crucial to the development of protective immunity in mice intravenously infected with *Mycobacterium tuberculosis*. J Exp Med (1997) 186(1):39–45. 10.1084/jem.186.1.39 9206995 PMC2198958

[100] Caruso AM Serbina N Klein E Triebold K Bloomand BR Flynn JL. Mice deficient in CD4 T cells have only transiently diminished levels of IFN-gamma, yet succumb to tuberculosis. J Immunol (1999) 162(9):5407–16. 10228018

[101] Mogues T Goodrich ME Ryan L LaCourseand R North RJ. The relative importance of T cell subsets in immunity and immunopathology of airborne *Mycobacterium tuberculosis* infection in mice. J Exp Med (2001) 193(3):271–80. 10.1084/jem.193.3.271 11157048 PMC2195922

