# Supplementary material for: De Novo Fatty Acid Synthesis During Mycobacterial Infection Is a Prerequisite for the Function of Highly Proliferative T Cells, But Not for Dendritic Cells or Macrophages
Source: Front Immunol. 2018 Apr 5;9:495. doi: 10.3389/fimmu.2018.00495 (PMC5895737; doi:10.3389/fimmu.2018.00495)
Supplement: Supplementary file 1 [file Presentation_1.PDF]

Supplementary Figure 1

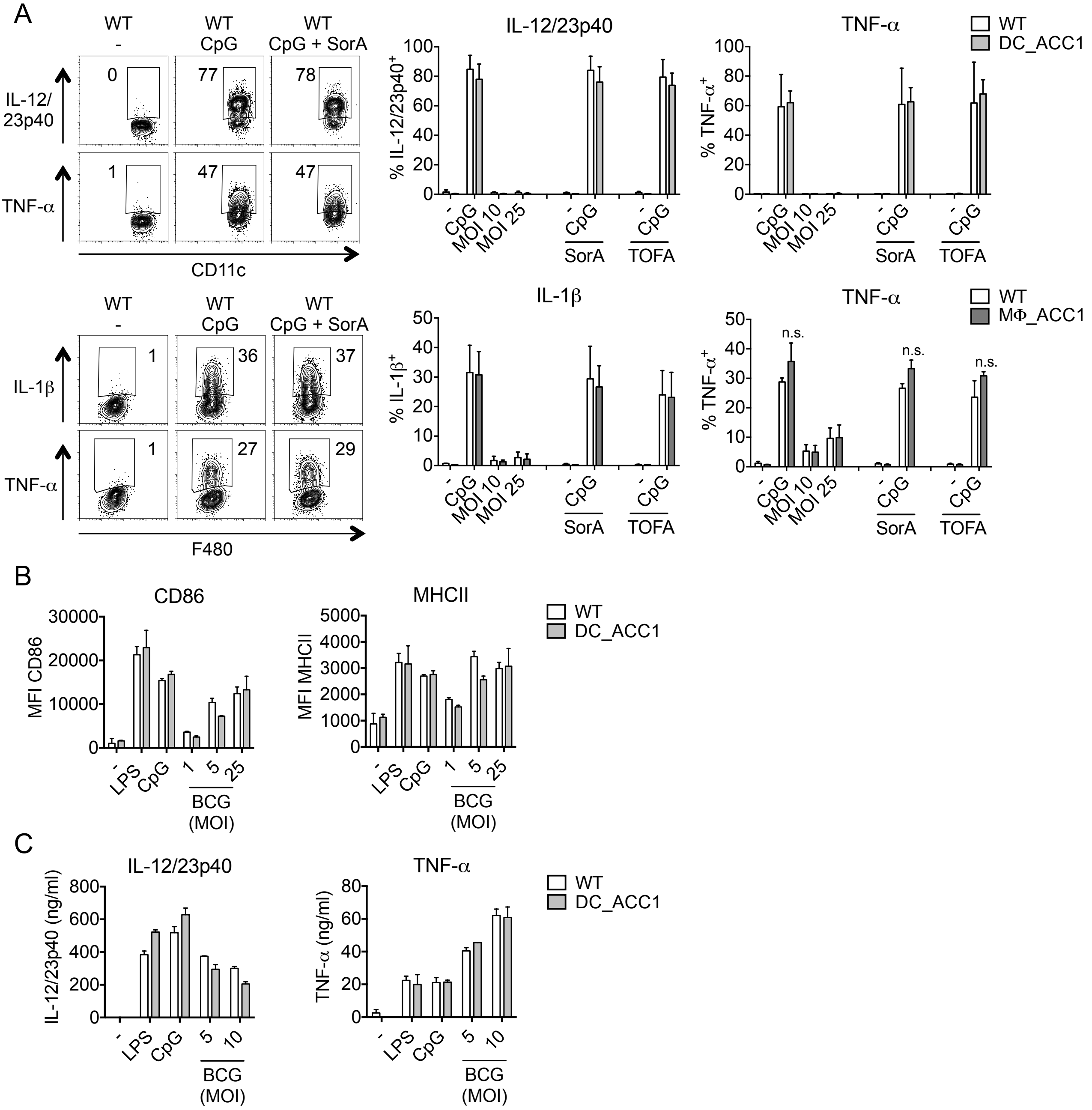

Suppl. Figure 1: ACC1 activity in DCs and macrophages is dispensable for inflammatory cytokine production and activation.

iCD103 DCs (A) or GM-CSF DCs (B, C) from WT and DC_ACC1 mice or BMDMs from WT and MΦ_ACC1 mice (A) were infected with different MOIs of *M. bovis* BCG and activation as well as cytokine production were determined. (A) Representative plots display cytokine production determined by flow cytometric analysis of intracellular cytokines after 6 h (left panel). Bar graphs display the frequencies of IL-12/23p40$^+$, TNF-α^+ and IL-1β^+ cells among live CD11c$^+$ or F480$^+$ cells (right panel). Results are displayed as mean ± SD of two pooled experiments out of four experiments. (B) Bar graphs display the MFI of CD86 and MHCII determined by flow cytometry after 24 h. (C) Bar graphs show the production of IL-12/23p40 and TNF-α measured by ELISA after 24 h. Results show mean of triplicates with error bars of SD (B, C) from one experiment (C) or as a representative of two individual experiments (B). n.s.: non-significant, two-way ANOVA with Bonferroni correction (A).

Supplementary Figure 2

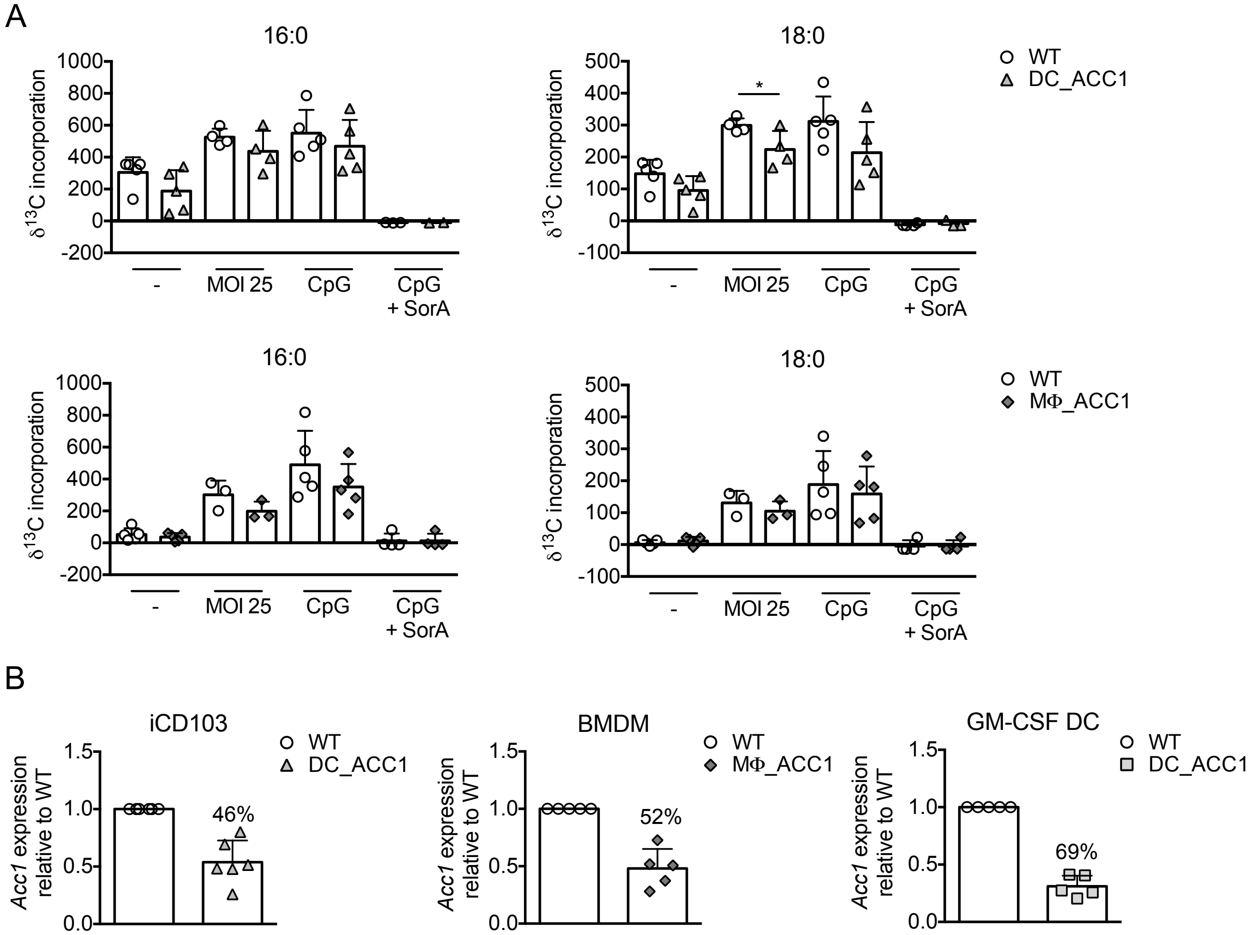

Suppl. Figure 2: Incomplete targeting of ACC1 in *in vitro*-generated cells results in partial inhibition of *de novo* FAS.

(A) iCD103 DCs from WT and DC_ACC1 mice (upper panel) or BMDMs from WT and MΦ_ACC1 mice (lower panel) were infected with *M. bovis* BCG and the rate of [U-$^{13}C_6$] glucose carbons into fatty acids was assessed ($\delta^{13}C$ incorporation). LPS and CpG served as positive controls. SorA was added together with CpG as a control of complete inhibition of ACC activity. Data are pooled from three to five different experiments. Each symbol represents an individual experiment. (B) The rate of ACC1 gene deletion was assessed by real-time PCR in iCD103 and GM-CSF DCs from DC_ACC1 mice and BMDMs from MΦ_ACC1 mice. Results are pooled from five (BMDMs and GM-CSF DCs) or six (iCD103 DCs) individual *in vitro* culture experiments. Error bars represent SD of mean. $^*P < 0.05$ and $^{**}P < 0.01$, $^{***}P < 0.001$, $^{****}P < 0.0001$, two-way ANOVA with Bonferroni correction (A).

Supplementary Figure 3

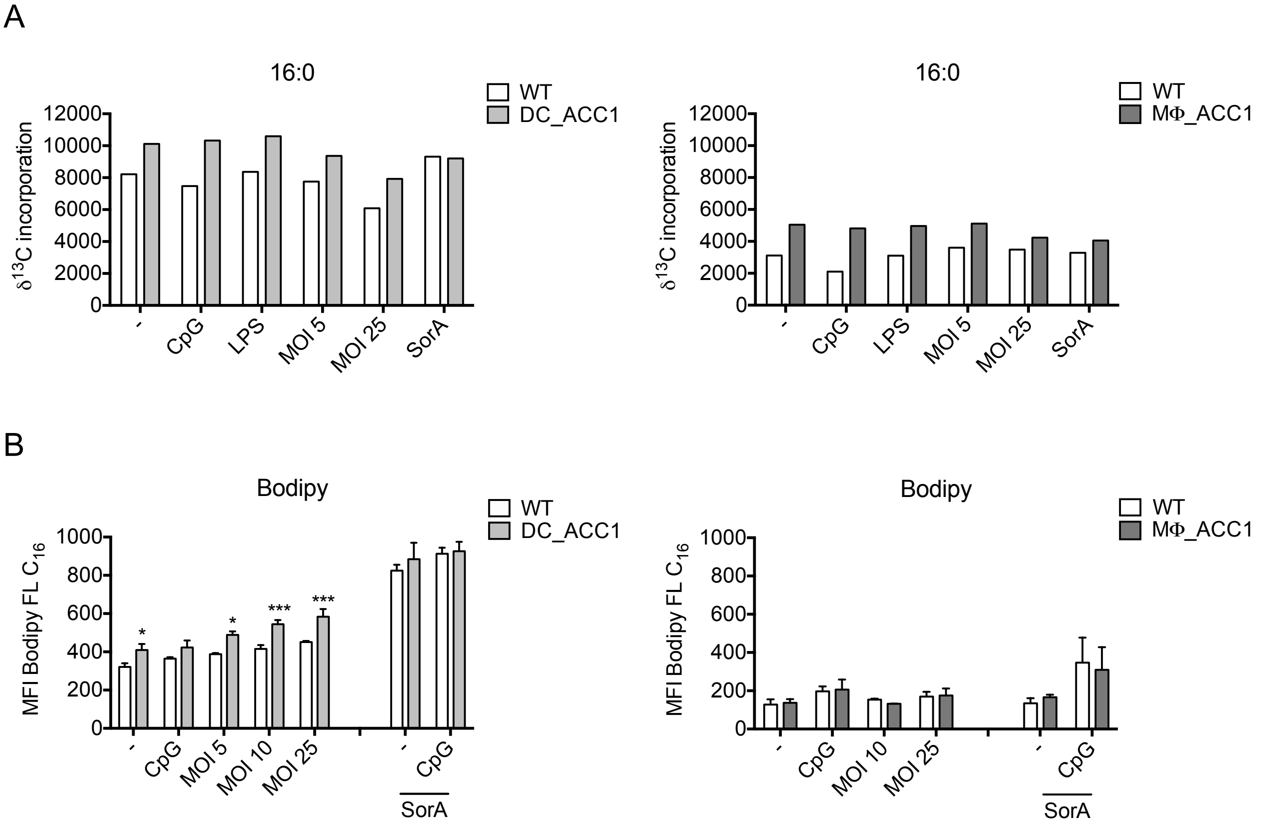

Suppl. Figure 3: Absence of ACC1 promotes the uptake of extracellular palmitate. iCD103 DCs from WT and DC_ACC1 mice or BMDMs from WT and MΦ_ACC1 mice were infected with different MOIs of *M. bovis* BCG and palmitate uptake was assessed. LPS and CpG served as positive controls. (A) Bar graphs show the incorporation ($\delta^{13}C$) of [U-$^{13}C_{16}$] palmitate into palmitate after 24 h of culture. Data are from one experiment with two pooled wells. (B) Bar graphs display the uptake of Bodipy FL C_{16} by iCD103 DCs and BMDMs at 24 h p.i. Results are representative of four experiments (iCD103 DCs) or pooled from two experiments out of three experiments (BMDMs) with triplicates each. Error bars represent SD of mean. *P < 0.05 and **P < 0.01, ***P < 0.001, ****P < 0.0001, two-way ANOVA with Bonferroni correction (B).

Supplementary Figure 4

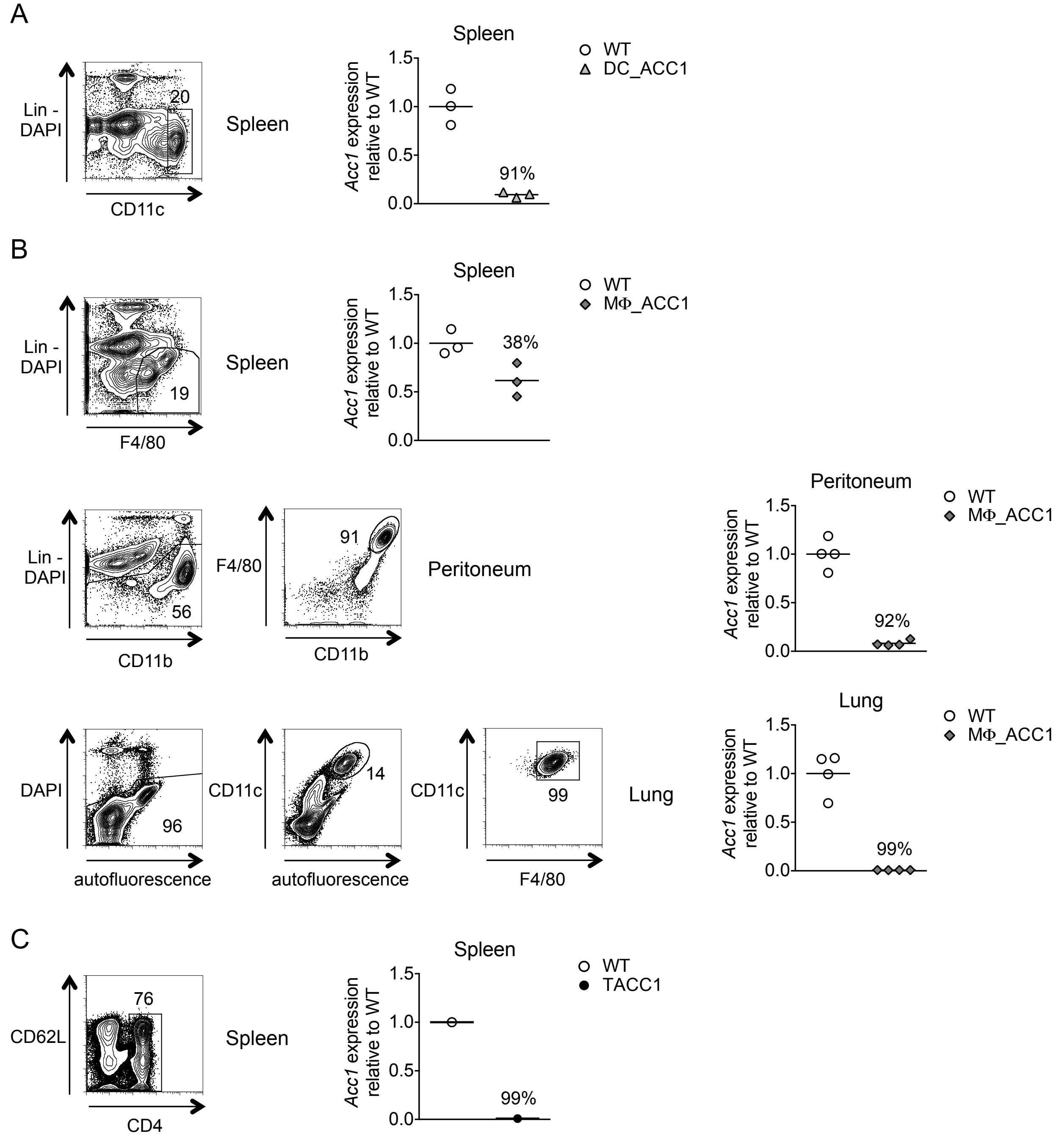

Suppl. Figure 4: ACC1 is efficiently targeted in DCs, macrophages and T cells *in vivo*.
DCs, macrophages or T cells were sorted from naïve WT, DC_ACC1, MΦ_ACC1 or TACC1 mice and the targeting efficiency of ACC1 was evaluated by real-time PCR. Representative flow cytometry plots display the gating strategy for sorting DCs (A), macrophages (B) and T cells (C) in the respective organs (left). Each symbol represents an individual mouse (right). Results are shown from one experiment (B: peritoneum and lung, C) or as a representative of two experiments (A, B: spleen) with n = 1-4 mice per group.

Supplementary Figure 5

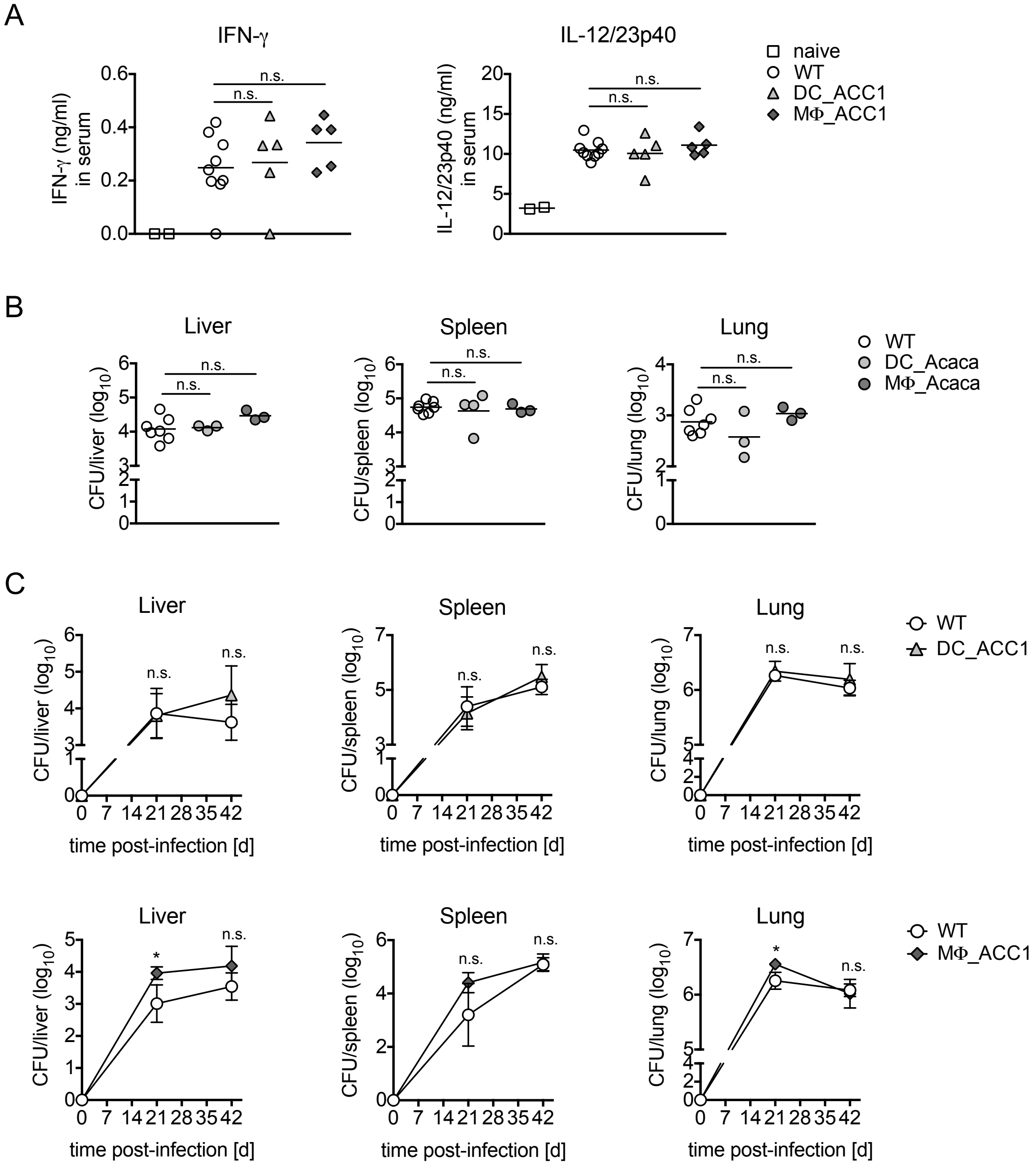

Suppl. Figure 5: ACC1 expression in DCs and macrophages is not a prerequisite for mycobacterial control.

(A) WT, DC_ACC1 and MΦ_ACC1 mice were infected i.v. with $2x10^6$ CFU of *M. bovis* BCG and on day 21 p.i. serum levels of IFN-γ and IL-12/23p40 were determined by ELISA. (B) DC_Acaca and MΦ_Acaca mice were infected i.v. with $2x10^6$ CFU of *M. bovis* BCG and the bacterial burden was determined in liver, spleen and lung on day 21 p.i. Each symbol represents an individual mouse. Results are shown from one experiment with n = 3-7 mice per group (B) or as a representative of three individual experiments with n = 2-9 mice per group (A). (C) WT, DC_ACC1 and MΦ_ACC1 mice were infected with a low dose of 100 CFU *Mtb* via the aerosol route and the bacterial burden was determined in liver, spleen and lung on day 21 and 42 p.i. Data represent mean ± SD from one experiment with n = 3-5 mice per group. $*P < 0.05$ and $**P < 0.01$, $***P < 0.001$, $****P < 0.0001$, n.s.: non-significant, one-way ANOVA with Dunnett´s correction (A, B) or Student’s t-test (C).

Supplementary Figure 6

A

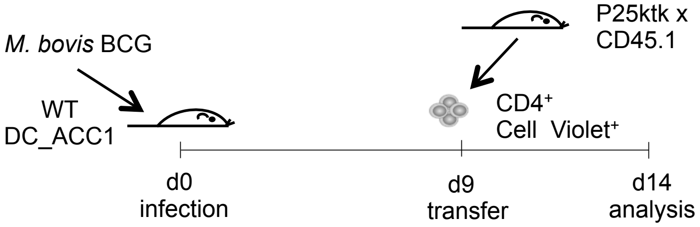

B

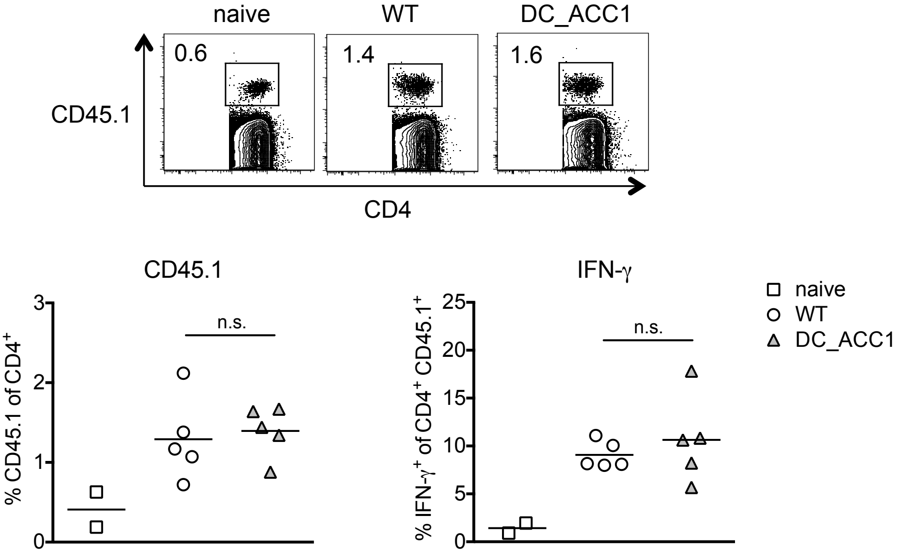

Suppl. Figure 6: ACC1 deletion does not impair DC priming capacity.
(A) Experimental scheme of the *in vivo* T cell priming assay. (B) Analysis of Cell Violet-labeled $CD4^+CD45.1^+$ P25ktk T cells five days after adoptive transfer into previously infected WT or DC_ACC1 mice. Representative flow cytometry plots display the frequency of adoptively transferred $CD4^+CD45.1^+$ P25ktk T cells in the spleen (upper panel). Graphs show the frequency of adoptively transferred $CD4^+CD45.1^+$ P25ktk T cells within total live $CD4^+$ T cells and the frequency of IFN-γ^+ production by these transferred cells upon re-stimulation with PMA/ ionomycin (lower panel). Each symbol represents an individual mouse. The results are shown as a representative of three individual experiments with n = 2-5 mice per group. *$P < 0.05$ and **$P < 0.01$, ***$P < 0.001$, ****$P < 0.0001$, n.s.: non-significant, Student's t-test (B).

Supplementary Figure 7

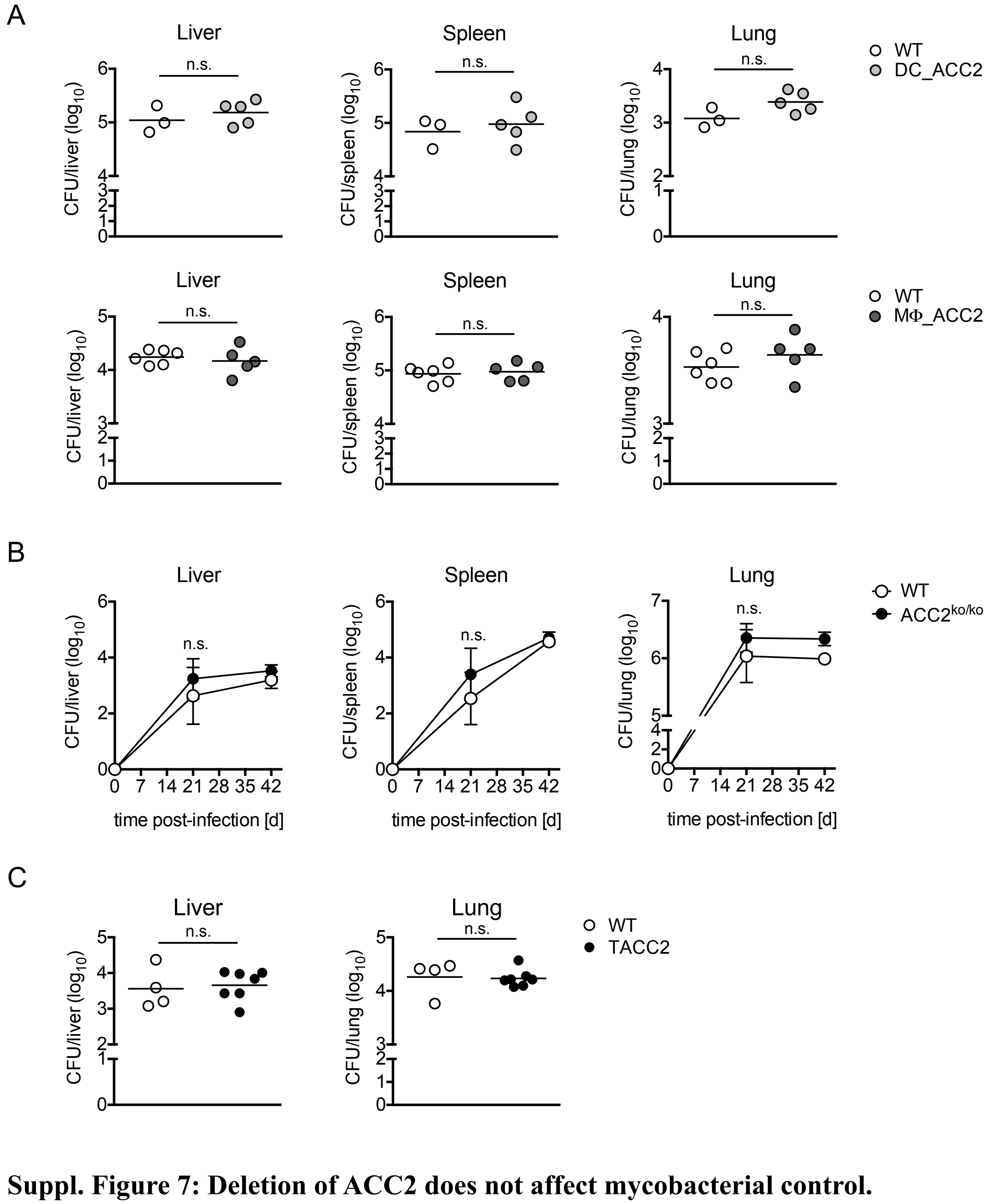

Suppl. Figure 7: Deletion of ACC2 does not affect mycobacterial control.
(A, C) WT, DC_ACC2, MΦ_ACC2 and TACC2 mice were infected i.v. with $2x10^6$ CFU of *M. bovis* BCG and the bacterial burden was determined in the respective organs on day 21 p.i. Each symbol represents an individual mouse. Results are shown from one experiment with n = 4-7 mice per group (C) or as a representative of two individual experiments with n = 3-5 mice per group (A). (B) $ACC2^{ko/ko}$ mice were infected with a low dose of 100 CFU *Mtb* via the aerosol route and the bacterial burden was determined in liver, spleen and lung on day 21 and 42 p.i. Data represent mean ± SD from one experiment with n = 2-4 mice per group. *$P < 0.05$ and **$P < 0.01$, ***$P < 0.001$, ****$P < 0.0001$, n.s.: non-significant, Student's t-test.
